# An Exploratory Eye-Tracking Study of Breast-Cancer Screening Ads: A Visual Analytics Framework and Descriptive Atlas

**DOI:** 10.3390/jemr18060064

**Published:** 2025-11-04

**Authors:** Ioanna Yfantidou, Stefanos Balaskas, Dimitra Skandali

**Affiliations:** 1 Department of Business and Management, Liverpool John Moores University (LJMU), Rodney Street, Liverpool L3 5UL, UK; 2 Department of Physics, School of Sciences, Democritus University of Thrace, Kavala Campus, 65404 Kavala, Greece; 3 Department of Business Administration, National and Kapodistrian University of Athens, 10679 Athens, Greece; dskandali@ba.uoa.gr

**Keywords:** eye-tracking, visual attention, time to first fixation (TTFF), dwell time, dwell dominance, visual analytics, public health advertising, breast cancer screening

## Abstract

Successful health promotion involves messages that are quickly captured and held long enough to permit eligibility, credibility, and calls to action to be coded. This research develops an exploratory eye-tracking atlas of breast cancer screening ads viewed by midlife women and a replicable pipeline that distinguishes early capture from long-term processing. Areas of Interest are divided into design-influential categories and graphed with two complementary measures: first hit and time to first fixation for entry and a tie-aware pairwise dominance model for dwell that produces rankings and an “early-vs.-sticky” quadrant visualization. Across creatives, pictorial and symbolic features were more likely to capture the first glance when they were perceptually dominant, while layouts containing centralized headlines or institutional cues deflected entry to the message and source. Prolonged attention was consistently focused on blocks of text, locations, and badges of authoring over ornamental pictures, demarcating the functional difference between capture and processing. Subgroup differences indicated audience-sensitive shifts: Older and household families shifted earlier toward source cues, more educated audiences shifted toward copy and locations, and younger or single viewers shifted toward symbols and images. Internal diagnostics verified that pairwise matrices were consistent with standard dwell summaries, verifying the comparative approach. The atlas converts the patterns into design-ready heuristics: defend sticky and early pieces, encourage sticky but late pieces by pushing them toward probable entry channels, de-clutter early but not sticky pieces to convert to processing, and re-think pieces that are neither. In practice, the diagnostics can be incorporated into procurement, pretesting, and briefs by agencies, educators, and campaign managers in order to enhance actionability without sacrificing segmentation of audiences. As an exploratory investigation, this study invites replication with larger and more diverse samples, generalizations to dynamic media, and associations with downstream measures such as recall and uptake of services.

## 1. Introduction

Public health communications increasingly use dense visual information to lead the public from awareness to action [1,2,3]. In this framework, where attention is first attracted and where it remains are pivotal to whether significant cues (e.g., screening eligibility, call to action, and credibility markers) are encoded and retrieved later. Much of the literature correlates root measures of eye movement with cognitive processes: time to first fixation (TTFF) captures initial salience, and fixation count and dwell time capture sustained information intake and effort [4,5,6,7]. Design evaluation research today again highlights that longer fixations are more likely to reflect more cognitive effort or interest, usually even confusion, and emphasizes the risk of interpretation of duration-based measures for task context.

Marketing and visual advertising research have employed eye tracking for over three decades to elucidate how attention to particular features (text, pictorials, and brand marks) predicts memory and persuasion impact [5,8,9]. Early research demonstrated that text and brand attention enhance memory for brands and that pictorial salience is able to attract attention and divert it to proximal copy. Later studies also establish that eye tracking actually measures visual attention to advertising accurately and that attention is a proximal determinant of effectiveness, with certain creative and context moderators intervening. First fixation effects are more complex: the first-hit location in itself is not informative for predicting choice but tends to lead later fixations to ultimately chosen alternatives; longer dwell and revisits are more powerful behavioral predictors of subsequent consequences [10,11].

In health communication, eye-tracking research has become more abundant on advertising and tobacco/e-cigarette warnings, repeatedly identifying children who pay more visual attention to prominent pictorials, individuals, and claims in ads than to dense text or warnings, processes with evident design implications for the building of screening appeals [4,12]. In e-cigarette advertising studies, to consider just one case, people’s pictures and other flavor descriptors capture earliest fixations, and promo/experiential slogans influence dwell; these patterns apply across user categories to a significant extent [4,9,11,13]. Systematic reviews of eye tracking on health warnings also report consistently low baseline attention to warning messages where design is not used to enhance salience. Such evidence suggests that transparent tools for making attention allocation transparent to creatives and policy advocates are needed.

Concurrently, persuasion in screening is still rooted in established communication theories. The Limited Capacity Model (LC4MP) foresees that there is limited processing capacity that is dynamically distributed to encode, store, and retrieve message features, thereby making visual competition in an advertisement salient in what is acquired [11,14,15]. Persuasion frameworks such as the Elaboration Likelihood Model (ELM) and Extended Parallel Process Model (EPPM)/Protection Motivation Theory explain when audiences elaborate central arguments (e.g., eligibility, efficacy) versus respond to peripheral cues (e.g., source, imagery) and how threat and efficacy must be balanced to motivate preventive behaviors such as mammography uptake. Design guidance from practice manuals (e.g., NCI’s Pink Book) likewise stresses clear calls to action, credible sources, and audience-tailored appeals [14,15,16]. Cumulatively, these traditions present a compelling argument that early capture and prolonged focus on high-value areas of interest (AOIs)—CTA, eligibility text, and trustworthy source—should be overt design objectives.

Existing public health advertising studies demonstrate that creative elements always steer attention toward emotionally engaging or promotional elements at the expense of actionability; campaign toolkits rarely supply visual diagnostics that are robust enough to compel micro-edits [3,16,17]. Through its emphasis on an atlas of breast cancer screening advertisements and a replicable visualization process, our research bridges theory and action, facilitating message design and testing of screening messages that more consistently engage and sustain attention to the content that generates behavior. Even with their robust measurement pedigrees, the vast majority of eye-tracking studies in advertising and health continue to center on point measures or basic AOI means, with relatively little concern for reproducible visualization pipelines and design-friendly descriptions that might be compared across creatives and target audiences [4,7,18]. Methodology reports always need explicit parameterization (e.g., fixation detection, smoothing kernels, normalization) and improved reporting, but comparability across studies is still challenging to attain, and designers are seldom given explicit visual summaries (e.g., atlas-style panels, first-hit maps, dwell-dominance matrices) corresponding to actionable edits [19,20]. As a result, benchmarking attention as pairwise “who wins more dwell” comparisons is seldom standardized, although such data crisply map onto the established paired-comparison frameworks [4,7,18,21].

To address these gaps, we present an exploratory eye-tracking atlas and visual analytics system for six breast-cancer screening commercials seen by women in the 40–60 age group. In place of onerous hypothesis testing, we prize standardized, replicable graphics and descriptions that may be applied immediately by creatives [20,22,23]. In particular, we (i) standardize a lean pipeline for per-participant AOI measures and ad-level summation; (ii) add first-hit and dwell-dominance aggregations that are comparable within and across ads; and (iii) offer subgroup splits (age, household, and education) to reveal audience-specific patterns to inform creative edits and media targeting. Methodologically, we (a) standardize aggregation-first displays (atlas-style small multiples, category overlays, and transition/precedence summaries) with transparent algorithmic reporting of best-practice choices available; (b) utilize paired-comparison reasoning in applying eye-tracking dwell (yielding interpretable dominance scores by AOI); and (c) present subgroup-aware tables/figures as reviewer-friendly baselines. Substantively, we provide design-ready findings for screening creatives—defining which pairs of imagery, copy, sources, and logos all consistently pull first and win dwell with this target audience.

In brief, the analyses reveal that early capture is strongly design-dependent—symbols and images are captured by first glance in image-driven designs, and emphasized headlines and institution badges attract attention to text and source indicators. The story is different with sustained attention: Throughout advertisements, message-carrying text and trusted sources more consistently attract attention than decorative imagery, with ad-specified exceptions. Audience halves evenly track the following movements: Family and older households enter through source/authority, highly educated readers through copy and places, and singles or youngsters through symbols and pictures. An “early-vs.-sticky” perspective translates these into editable movements—moving late-but-sticky CTAs up towards probable entry points and streamlining early-but-not-sticky items—while in-house testing and adhering to traditional dwell measures validate the diagnostics’ strength. Together, the atlas provides a nuts-and-bolts reproducible model for public health creatives such that the most fundamental elements capture attention first and hold it long enough to spark awareness and action.

The remainder of this paper is organized as follows: Section 2 discusses the literature on early vs. sustained attention to advertisements and health messages, eye-tracking visualization conventions, and descriptive vs. inferential approaches to public health creatives. Section 3 outlines stimuli, participants, apparatuses, AOI definitions, and analysis pipelines, including first-hit/TTFF measures, tie-aware dwell-dominance matrices, and atlas visualizations. Section 4 presents the results for RQ1–RQ3: early capture patterns; pairwise dominance as sustained attention; subgroup shifts by household, age, and education; and overall “early-vs.-sticky” diagnostics combined. Section 5 offers sanity checks and robustness tests, including internal matrix properties and concordance with traditional dwell summaries. Section 6 distills design heuristics and practical implications for policymakers, campaign managers, and educators, recasting the atlas as fix-it guidelines. Section 7 ends with contributions, limitations, and future research directions.

## 2. Literature Review

### 2.1. Early vs. Sustained Attention in Advertising and Health Messages

Eye-tracking studies differentiate between early attention capture (most frequently quantified as time to first fixation or TTFF) and prolonged processing (cumulative fixation count or dwell time on an item) [2,24,25,26]. They are measures of distinct cognitive processes: TTFF is a measure of an item’s efficiency in attracting attention, while dwell time is a measure of its efficiency at holding attention for further processing. In visual advertising, the audience generally concentrates on prominent images (e.g., faces or photographs) ahead of text; for instance, Pieters et al. [27] described how participants “consistently viewed the image before addressing textual information”. Early fixations are controlled by salience and structure, but longer fixations are associated with persuasion and encoding. Indeed, traditional research shows that longer inspection of brand products or health messages enhances recall of them.

In print advertising, heightened preoccupation with the brand logo is predictive of heightened recall of the brand, and in health warning announcements, even short text warnings are enhanced by longer viewing duration: Research has defined positive relationships between overall warning-label fixation duration and subsequent warning-content recall [4,7,18,28]. In contrast, items that never receive a fixation have a minimal chance of being remembered. Initial tests of tobacco and alcohol adverts demonstrated that text-only warnings are frequently totally nonvisible (in ~40% of experiments) or take up just 5–8% of visual attention and lead to poor recall unless salience is enhanced by adding color or a picture. These results emphasize the significance of first hit and dwell: a main health message must be caught with a glimpse and viewed for long enough to be processed [2,7,26].

However, evidence is mixed on which matters more [7,24,26]. Several works show that fixations early on are not enough to guarantee persuasion or choice, e.g., having control over what one sees first has limited ability to modify end preferences, and that prolonged attention and re-attention (multiple fixations) is a more reliable predictor of results such as brand recall or risk comprehension [26]. With respect to screening commercials, then, this implies that a text- or source-cued call to action must not only capture viewers’ attention initially but also “stick” in their eyes to support recall and guide intentions. Surprisingly, recent eye-tracking experiments with tobacco warning labels illustrate how control of content directs attention patterns: In a study, warnings for less familiar risks of smoking caused observers to point the text sooner and longer, and known risk warnings elicited more looking to the image—but overall warning information recall was not varied by condition [29,30]. This implies that first fixation vs. dwell effects might be content-dependent and not necessarily linearly dependent on memory, supporting the justification for a qualitative, descriptive comparison between both measures in exploratory studies like ours.

Current research on encouraging breast-cancer screening emphasizes the value of an exploratory, design-focused eye-tracking atlas for practice [6,8,27]. Criticisms of theory contend that standard mammography interventions under-emphasize social context and cultural diversity at the expense of individual cognitions (e.g., HBM); culture-based, multilevel approaches are essential—a recommendation for our subgroup-sensitive analyses and design heuristics. Social marketing interventions similarly demonstrate that adoption depends on system and message levers (e.g., doctor referral, access barriers, awareness of guidelines), suggesting multicomponent solutions that combine communication remedies with service delivery—exactly the kind of certain changes our atlas will coordinate (e.g., relocating the CTA and source cues up and forward and making them “stickier”) [31,32,33]. Evidence from a large comparative campaign experiment attests to the combined functions of mass media and social networks in creating awareness and large between-area variation in knowledge and source to emphasize the necessity of audience segmentation in creative testing. Simultaneously, “pink ribbon” advertising critiques caution in that persuasive signals (color/ribbon/hope frames) replace information content (eligibility and how to behave), risking creating an “illusion of knowledge”; our emphasis on first hit and dwell on AOIs like CTA, eligibility copy, and trusted source actively bridges that gap. Widespread reviews agree on effective strategies, reduced anxiety, self-care training, and reminder systems being the effective factors in improving screening activity, while systematic reviews yield net positive effects of health-promotion interventions but inconsistent study quality, highlighting the importance of open, reproducible analyses that can be sketched at a glance by practitioners.

### 2.2. Visual Analytics of Eye-Tracking: Heatmaps, AOIs, and Scanpaths

With the increasing application of eye-tracking in HCI and marketing, solid methodological recommendations are available on how to visualize and summarize eye movement data [21,24,25]. Heatmaps are a common workhorse for displaying collective group attention to static stimuli; conventions dictate smoothing raw fixations by a Gaussian blur of roughly one degree of visual angle to simulate foveal vision and normalizing densities (e.g., 0–1) for comparison. It should be noteworthy that such parameters are listed, since changes in smoothing or in fixation detection may modify outcomes. For routine fixation detection, the standard algorithms I-DT (dispersion-threshold) and I-VT (velocity-threshold) are applied to analyze eye movement streams, with thresholds typically tuned to find a balance between sensitivity and noise (e.g., a spatial tolerance ~1° for dispersion) [2,7,25]. Areas of interest (AOIs) enable quantitative comparisons between them (e.g., dwell time within an AOI, percentage of viewers who viewed a specific AOI). However, new research warns that it is possible to introduce variation when operationalizing AOIs: reanalysis of several experiments found that variation in the size or shape of AOIs can sometimes affect statistical results, and simulation illustrates complex nonlinear relationships between AOI features and tracker performance [18,21].

Since definitions of AOIs and tracking accuracy both affect whether an element is marked as “seen,” hit report rates also vary with the definition of regions and measurement resolution. For increased reproducibility, recommendations now cover complete transparency regarding stimuli, AOI definitions, and processing parameters, so results can be relied upon and replicated [4,18]. With these suggestions in mind, we implement an entire visualization pipeline to capture and report results in an atlas of side-by-side small multiples by ad and audience subgroups compared to a single pooled heatmap. This is perfectly suited to direct comparisons between populations and creatives, a typical weakness of past attempts at eye tracking. Complementary HCI studies validate comparative visualizations; for example, Hooge et al.’s [34] arrow plot aggregates scanpaths by AOI transition diagrams and provides them with summary metrics (e.g., scanpath entropy to indicate interobserver agreement and T50, the duration taken for 50% of observers to view a target) in an effort to describe how designs control an individual’s gaze. Extending that argument a step further, we introduce not only static heatmaps but also transition patterns and rank-order attention metrics and incorporate a qualitative atlas, uncovering design-significant differences—for instance, whether the headline always appears before the image in one advertisement and is absent in another—that are lost with mere summary statistics.

Visual inspection here is optimized to the requirements of diagnosing PSAs used in screening for breast cancer, where the design task is to ensure that eligibility/benefit text, call-to-action/website, and credible sources are noticed first and held long enough to be coded [4,18]. Consequently, we organize AOIs into six message-based categories—Text, Image/Visual, Symbol (such as ribbon, icons), Logo, Website/CTA, and Source/Authority—so attention can be specified in terms relevant to public-health creatives (e.g., “CTA” and “Source” performance, not generic regions). Heatmaps are utilized in an effort to report subgroup-level focus on every static advertisement; we utilize a Gaussian filter (≈1–1.5° of visual angle) and normalize intensity levels to [0, 1] to make maps scalable between ads and subgroups. Since smoothing, fix detection, and threshold selections could have an impact on visible “hotspots,” we supply these parameters and retain all per-participant × AOI summaries and maps [4,21,28].

To make comparisons actionable on the level of individual creatives, we deliver results as an atlas of small multiples: one panel per ad and per audience stratum (age, household, and education). This layout allows one to see at a glance whether an empowerment-motif poster, for example, attracts initial fixation to a face but loses sight of the CTA, while a statistics-based poster will attract extended dwell on text but will not attract initial fixation. In addition to static heatmaps, we calculate rank-order and pairwise aggregates that inform our RQs directly. Since screening campaigns must appeal to diverse audiences, we look at subgroup deltas (subgroup − overall) in first-hit% by category as small heatmaps, finding where, for example, Text receives proportionally more first fixations between 46–50 and 40–45, or where Symbols underperforms on single-parent families. To combine early capture and stickiness into one diagnostic, we put an “early-vs.-sticky” scatter per ad (x: first-hit%; y: dominance score) on top of each other. These expose patterns with explicit design implications: early-but-not-sticky CTAs indicate increasing contrast or proximity to the early hotspot; sticky-but-late sources indicate repositioning or pre-attentive cues to capture the first fixation. Collectively, these breast-cancer-specific visualizations—heatmaps, dominance matrices, small-multiple subgroup panels, and early-vs.-sticky plots—form a reproducible descriptive atlas that explicitly fulfills our exploratory objective and RQs, interpreting raw gaze streams into design-ready hypotheses for screening ad improvement.

### 2.3. Descriptive vs. Inferential Approaches in Neuromarketing

The majority of the latest neuromarketing studies depend on inferential tests to investigate hypotheses (for instance, whether image X’s warning will produce longer dwell and improved recall compared to image Y). In applied and exploratory settings, however, descriptive analytics and visualization play a similar role [24]. Our method follows consumer neuroscience best practice in deriving design-ready insights from attention data drawn from moderate samples [2,7,24]. In practice, eye-tracking outputs (heatmaps, gaze plots, and “attention scores”) are typically used diagnostically to guide creative decision-making and not formally testing for significance. The reasoning is that formative testing looks for hypothesis generation and heuristics—learning what attracts or loses audience attention—rather than establishing generalizable effects. Previous research on health communication also blends techniques: Chen-Sankey et al. [4] present statistical comparisons with “exploratory, descriptive analyses” of eye-scanning between novel warning labels, essentially comparing stimuli for interest or disgust. To facilitate such comparisons adequately, we call upon paired-comparison logic.

We introduce a dwell-dominance measure between ads that can be interpreted through Bradley–Terry models applied to pairwise preferences [25]. Informally, when one ad’s key AOI consistently receives longer looks than the other’s, we tally that as a “win” in a paired competition—just as Thurstone’s comparative judgment in psychophysics [25]. This provides a natural ranking of creatives by visual impact, augmenting conventional ANOVA or regression. We highlight the exploratory nature of this study: The goal is to reveal patterns (e.g., Ad A’s call to action beating Ad B’s attention capture) and provide visual proof for design, not to make population inferences at large. Inferential testing is still suitable for theory testing or preregistered hypotheses, but with our sample (N ≈ 30) and campaign-specific interest, a descriptive visual analytics approach is most appropriate [25,34,35]. This position aligns with best practices requiring that eye-tracking must inform not only hypothesis testing but also practitioner-oriented, actionable insights.

Cumulatively, the research so far demonstrates that both first-fixation and dwell-time measures are predictive of ad effectiveness; that meticulous visualization and AOI-based summaries reveal more fine-grained cross-stimulus discrimination; and that exploratory, descriptive research can provide actionable direction in neuromarketing applications. Large gaps remain, however. Standardized visualization procedures for comparing multiple ads are uncommon, and pairwise attention comparisons (for creative ranking or variants) are seldom, if ever, provided despite their face validity for design. In addition, subgroup differences in visual attention (i.e., how demographic groups look at breast-cancer screening advertisements) are understudied, since most research focuses on overall means. Our research fills these gaps by presenting a descriptive atlas of attention across six screening advertisements (with AOIs over key message elements), developing pairwise dwell-dominance measures based on comparative-judgment theory, and examining subgroup attention patterns—thus offering design-ready input to health communication campaigns. The relevant work, therefore, mirrors our emphasis on an exploratory visual analytics strategy that is methodologically open and practical-impact-focused; thus, the following research questions were formulated:

- ***RQ1 (Early Capture)****: Where does attention land first—by AOI and semantic category—and how concentrated is initial capture within each ad? (first-hit percentages and entry ranks).*
- ***RQ2 (Sustained Attention)****: Which elements dominate dwell when contrasted pairwise within an ad (dwell-dominance matrices summarizing P[AOIi>AOIj])?*
- ***RQ3 (Audience Shifts)****: How do early capture and dwell patterns shift across strata (age, household, and education)?*

## 3. Research Methodology

### 3.1. Design, Apparatus, and Participants

We carried out a within-subject lab experiment with eye-tracking measurements of visual attention to six static breast cancer screening advertisements (public service announcements). Design was within-subject: All six ads were viewed by everyone. Order effects were controlled through per-participant counterbalancing where possible (balanced Latin square) or otherwise by randomization [36,37]. All advertisements appeared full screen for 10 s and included a 5 s neutral gray screen to restore gaze and minimize carryover, as per controlled exposure regimes [38,39]. The trials were conducted at the Laboratory of Integrated Marketing Communications of the National and Kapodistrian University of Athens in conditions of constant ambient lighting and low levels of disturbance.

Eye gaze was tracked with a Tobii Pro Nano (60 Hz), enabling chinrest-free viewing with natural head movement [40]. A 9-point calibration/validation was carried out at ~60 cm viewing distance at the session’s commencement, with repeated recalibration as required. Stimuli were presented full-screen on a 24″ LCD at 1920 × 1080 px (active area ≈ 53 × 30 cm). At ~60 cm, 1° of visual angle corresponds to ~1.0–1.1 cm. Facial expression analysis (iMotions) was tracked but not analyzed for the purposes of this research. The equipment used is consistent with best practices in visual attention and neuromarketing research.

We employed quota-stratified purposive sampling to enlist 40–60-year-old women—the ideal target population for promoting screening [36,37,39]. Thirty were enlisted through university mailshots, local clinics, social media, and word of mouth. Quotas were employed to sample heterogeneity on the following: (i) age bands (40–45, 46–50, 51–55, and 56–60), (ii) type of household (married with/without children; single with/without children), and (iii) education (compulsory/secondary; university; postgraduate). On arrival, the participants filled in a standardized briefing, signed written informed consent forms according to GDPR and institutional ethics regulations, and answered a short demographic questionnaire used for exploratory subgroup analyses (age, household, and education).

Overall, 30 respondents were thus employed as the final sample (Table 1). Due to heterogeneity considerations, three demographic dimensions were quota-set. Initially, age was evenly divided into four groups, with majority being in the 40–45 and 56–60 (30.0% each), 51–55 (23.3%), and 46–50 (16.7%) groups. Second, most of the participants were married with kids (63.3%), 16.7% were singles without kids, and 10.0% were singles with kids and married without kids. Third, education-wise, 43.3% reported they completed mandatory high school, 23.3% reported they had a university degree, 20.0% reported they had a master’s or above, and 13.3% reported primary school as their highest level of schooling.

### 3.2. Stimuli and Areas of Interest (AOIs)

Stimuli were six professionally designed static ads: Three were ongoing public-health campaigns, and three were ad hoc created by the creative agency DIMANA. For component-level analysis, human-annotated AOIs were manually drawn in Tobii Studio with human annotation guidelines [36]. In total, 41 AOIs were annotated across the set and coded to six semantic classes: Text, Image/Visual, Symbol, Logo, Website/CTA, and Source/Authority. AOI boundaries were set for each ad once for all participants. For descriptive layout covariates, we extracted the AOI pixel area and eccentricity (screen center distance). We assigned AOIs with a standard naming scheme (Ad3_SmallText2) such that clean merging could be carried out with the gaze stream and subgroup variables (Table 2).

**Figure 1:**
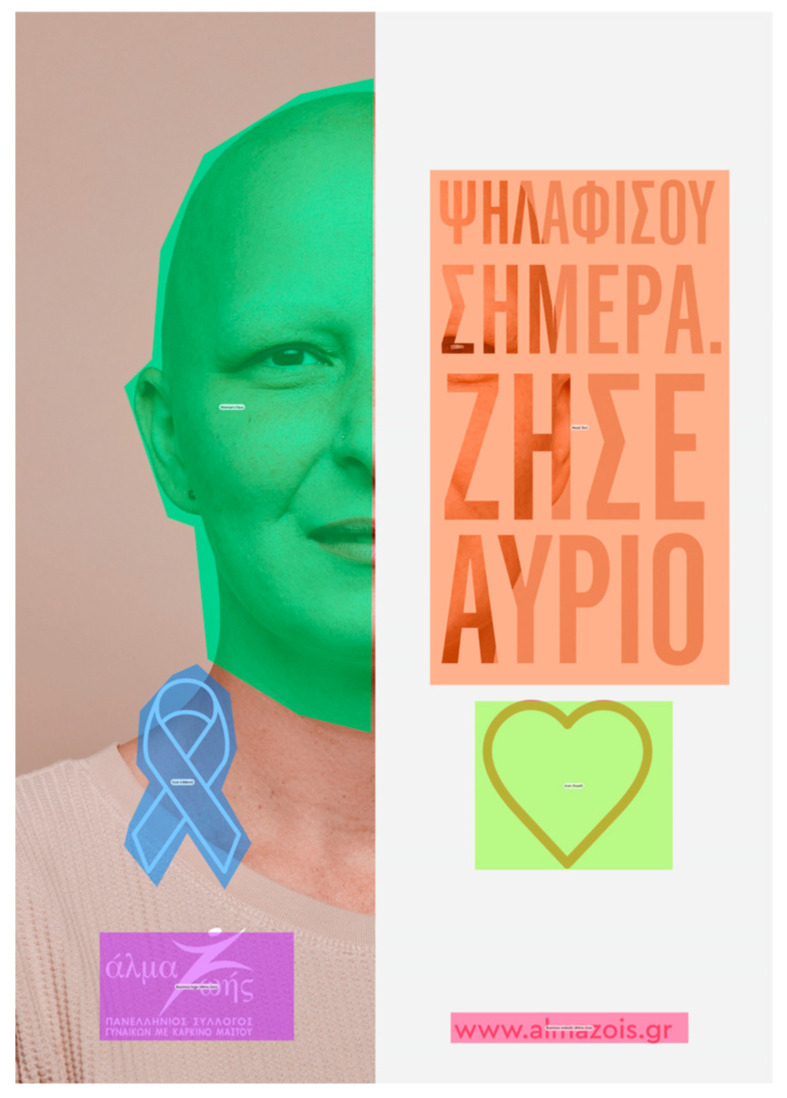
Example AOI delineation on a representative breast cancer awareness ad with a message of hope and survival after treatment (English translated caption: “Do a self-exam today. Live tomorrow.”). Colored polygons denote AOIs by category (Text, Image/Visual, Symbol, Logo, Website/CTA, and Source/Authority). Overlap priority is Website/CTA > Logo > Symbol > Source/Authority > Text > Image/Visual, ensuring that boundary fixations are assigned consistently. Polygons were drawn at native resolution in Tobii Studio and used for all AOI-based metrics (TTFF, fixation count, and total fixation duration).

AOIs were delineated as closed polygons at native stimulus resolution within Tobii Studio and checked at 200% zoom to confirm edge accuracy. We did not delineate items smaller than text size and did not delineate clean decorative whitespace. Polygon vertices (pixels), AOI ID, semantic category, and ad ID were exported and merged 1:1 with the gaze stream to facilitate AOI-based metrics (TTFF, number of fixations, and total fixation duration). Where AOIs overlapped or touched, we used a static overlap-priority rule—Website/CTA > Logo > Symbol > Source/Authority > Text > Image/Visual—so boundary fixations were always attributed to the most actionable/diagnostic object. Ties within dwell-dominance calculations were left as 0.5, and support (N < 5) cells were eliminated from visualizations (not in algebraic checks). Table 2 lists the six categories and their definitions, along with examples that were employed in annotation. To make AOI delineation transparent, we provide an illustrative overlay on a representative stimulus (Figure 1).

**Figure 2:**
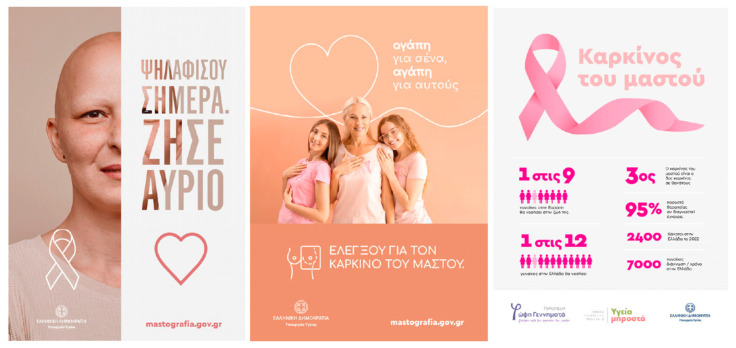
Breast cancer awareness posters presented to participants. Left to right: poster with a message of hope and survival after treatment (**left**; English translated caption: “Do a self-exam today. Live tomorrow.”); poster with a message of regular screening and self-exam (**middle**; English translated caption: “Love yourself, love them. Get screened for breast cancer.”); infographic-style panel presenting prevalence data and early-detection information (**right**; English translated caption: “Breast Cancer: 1 in 9 women will develop breast cancer during her lifetime. 3rd leading cause of cancer death among women. 95% survival if detected early. 1 in 12 women in Greece have not had a mammogram. 2400 new cases annually in Greece. 7000 women live today after breast cancer treatment.”).

**Figure 3:**
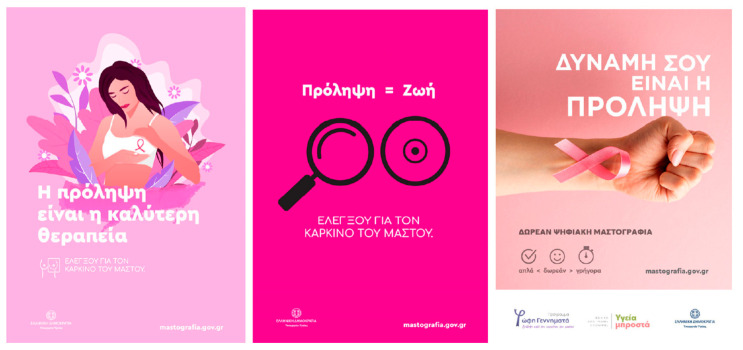
Breast cancer awareness posters handed out to the participants. From left to right: Artwork highlighting prevention as the key (**left**; English translated caption: “Prevention is the best treatment. Get screened for breast cancer.”); graphic design in which a magnifying glass symbolizes screening and detection (**center**; English translated caption: “Prevention = Life. Get screened for breast cancer.”); empowerment poster encouraging prevention and utilization of free mammography programs (**right**; English translated caption: “Your strength is prevention. Free digital mammography.”).

Together, the combined comparative design layouts—survivor narrative, family background, fact messaging, iconography of self-reflect, effective color/objectification test, and empowerment/access info—systematically controlled attention-relevant factors (imagery vs. text, symbols, logos, website/CTA, and source/authority). Within-condition variation was used for attention contrasts at the AOI level (TTFF, fixation frequency, and fixation duration) and to determine if 40+-year-old women differentially draw and sustain attention to sources of emotions, texts, symbols, and institutions, with an AOI map example provided in Figure 1 and the full stimulus set provided in Figure 2 and Figure 3.

### 3.3. Procedure and Measures

The participants saw the six static ads in a pre-defined order: each stimulus for 10 s and a 5 s mid-gray inter-stimulus screen. Gaze was tracked continuously and broken down into three standard area-of-interest (AOI) metrics per participant × ad × AOI: (a) time to first fixation (TTFF, ms)—latency from stimulus onset until the first fixation in the AOI, indexing early capture or pre-attentive salience; (b) fixation count (FC, number of fixations)—number of returns to the AOI, indexing revisiting, and processing episodes; and (c) total fixation duration (FD, ms)—total time fixated within the AOI, indexing depth of processing/engagement [36,37]. AOIs were determined a priori and were the same for all participants and corresponded to the semantic categories employed in this paper (Text, Image, Symbol, Logo, Website/CTA, and Source/Authority).

Fixations were identified with Tobii’s I-VT classifier (velocity threshold ≈ 30°/s), using a minimum fixation duration of 60–80 ms and standard post-processing to merge adjacent fixations within 0.5° and ≤75 ms. Blinks and invalid samples were removed. Trials were excluded when valid gaze samples fell below 70%, when calibration drift exceeded ~1° of visual angle, or when the participant visibly disengaged from the screen [41,42]. For AOIs that never fixated within the 10 s window, TTFF was right-censored at 10,000 ms; FC and FD were zeroed for non-fixated AOIs [43,44]. All responses were saved in “long” format (participant × ad × AOI) to facilitate atlas visualizations (heatmaps, small multiples, and early-vs.-sticky plots) and descriptive aggregations (e.g., first-hit percentages, dwell-dominance matrices). In accordance with our reporting elsewhere in the paper, low-support cells (e.g., <5 overlapping viewers for a pairwise comparison) were excluded from figures but kept for completeness in summary tables [45,46].

Following exposure, the participants rated brief post-exposure (credibility, persuasiveness, emotional impact, and motivation). Self-ratings are recorded for transparency purposes but are not considered secondary to ongoing visual analytics objectives and are not inferentially tested here.

### 3.4. Data Processing and Visual Analytics Protocol

All analyses are performed at the participant × ad × AOI level from a clean long-form table. AOI labels are tokenized to ad and aoi_name, and their semantic types are normalized to six categories (Text, Image/Visual, Symbol, Logo, Website, and Source/Authority). We keep time to first fixation (TTFF, ms), fixation count (FC), and total fixation duration (FD, ms) and also participant’s age, household, and education strata per observation. Within the ad subject, AOIs are ordered by TTFF to determine the order of entry; the lowest TTFF determines the first-hit AOI of a trial [47]. For an AOI that receives no fixation within the 10 s presentation, TTFF is right-censored at 10,000 ms and is not used for determining the first hit for the subject, but it is added to latency summarization.

We abbreviate initial processing and capture with ad-specific distributions. The first-hit percentage estimates the percentage of subjects whose first fixation fell upon a specific AOI, with a corresponding summary at the category level. Latency is quantified through the median TTFF by AOI and by category to show how rapidly items draw gaze. Processing is equated by FC and FD, reported as medians with interquartile ranges. These figures are added up into a concise “benchmark” table by ad, a reviewer-convenient reference that all downstream visualizations are based on.

#### Pairwise Dwell Dominance (Key Analytic)

In order to allow us to compare objects in an ad on some common basis, we carry out tie-aware pairwise comparisons of dwell time [41,44,48]. For each ad for each AOI pair (i, j), we restrict to subjects who fixated on both AOIs and estimate the probability that AOI i receives more dwell than AOI j, with ties counted as half. The matrix P obtained has $P_{ii}=0.5$ and $P_{ij}+P_{ji}=1$ by construction.

We record the support $N_{ij}$ as the number of participants contributing to each pair; low-support cells (default *N* < 5) are masked in figures but retained for transparency in accompanying tables. A dominance score for each AOI is then defined as $s_{i}=\;2\left(\bar{P_{i}\;}-0.5\right)$, yielding an $s_{i}\in \left[-1,1\right]$ index that ranks “winners” (positive values) and “losers” (negative values) within the ad [44,48]. The same procedure is repeated at the category level after aggregating dwell within the category per participant, producing a cleaner, high-level ranking that complements AOI-level results.

Heterogeneity of the audience is described by examining descriptively redundant first-hit percentages at the category level by Age, Household, and Education strata. For each ad, we then construct delta matrices by the overall first-hit percentage minus the subgroup value, which provides percentage-point differences that indicate whether an audience in question is hearing comparatively more or less from a category. Such deltas are shown as small-multiple heatmaps, emphasizing the most significant directional changes by principal text, and entire matrices are itemized in Section 4.4.

Matrix properties are checked for every ad for numerical coherence: $P+P^{⊤}$ top is an all-one matrix in tolerance, and the P diagonal equals 0.5. AOI dominance scores are cross-correlated against the median FD per ad via Pearson and Spearman coefficients to confirm concordance between pairwise dominance and strict processing time. Coverage is represented in support-N heatmaps so masked regions can be understood in light of available evidence [44,45,47]. Missingness and censoring are dealt with explicitly as outlined above, and all summaries are augmented by underlying counts (pairs or participants) in order to place the stability of estimates into context.

Every advert is shown as an atlas panel consisting of a dwell-dominance heatmap (AOI level) reorganized by dominance, a small bar chart of the top five AOIs by dominance score, and a companion category-level dominance heatmap. To identify the early capture and sustained attention association, we again graph an “early-vs.-sticky” scatter with each AOI’s first-hit rate on the *x*-axis and its dominance score on the *y*-axis so that we can easily ascertain which AOIs attract attention early but are not sticky (and vice versa). Subgroup effects are indicated in the form of delta heatmaps for Age, Household, and Education. Lastly, benchmark tables reporting the median TTFF, FC, and FD with interquartile range and frequency precede figures to enable reproducibility and applied analysis.

## 4. Data Analysis and Results

### 4.1. Preliminary Analysis

An overall aggregated table of eye-tracking measures was formed for measuring visual attention in all pre-defined areas of interest (AOIs) quantitatively [36,37]. The most critical eye-tracking measures were time to first fixation (TTFF), fixation count, and total fixation duration, which represent various attentional salience, cognitive processing, and engagement metrics. Each AOI received these measures, which were segregated by advertisement and semantic AOI type (i.e., Text, Symbol, Image/Visual, Logo, Website, Source/Authority). The overall analysis of the gaze behavior of all identified areas of interest (AOIs) offers comparable and theory-supported patterns in which participants visually processed the six breast cancer screening ads. The data were composed of 41 AOIs in six semantic categories—Image/Visual, Text, Symbol, Logo, Website, and Source/Authority—each with three basic eye-tracking measures drawn for the analysis: time to first fixation (TTFF), fixation count (FC), and fixation duration (FD) (Table 3).

The overall analysis of the gaze behavior of all the identified areas of interest (AOIs) offers comparable and theory-supported patterns in which participants visually processed the six breast cancer screening ads. The data were composed of 41 AOIs in six semantic categories—Image/Visual, Text, Symbol, Logo, Website, and Source/Authority—each with three basic eye-tracking measures drawn for the analysis: time to first fixation (TTFF), fixation Count, and fixation duration.

Among the categories, Website components were most rapidly identified at 133.82 ms (TTFF), followed by Source/Authority indicators at 165.64 ms and Logos at 219.83 ms. This suggests that visually salient categories such as campaign URLs, government endorsement, or logos are recognized early during scanning because they possess standard form, spatial predictability, or high contrast. However, whatever initial visual registration takes place is not necessarily revealed in processing depth or subjective salience, particularly if features of this kind are spatially or semantically remote from the center of the ad narrative.

In terms of fixation duration, which indicates the degree of cognitive and affective processing invested in a visual feature, Logos had the longest mean viewing duration (314.07 ms), followed by Symbols (302.70 ms) and Source/Authority cues (275.77 ms). These features can attract prolonged attention because of their symbolic content (e.g., pink ribbons, organizational logos) or the interpretive work needed to be coaxed into alignment with the message. This means that even though they may not draw the eye initially, they do draw mental attention once they have been processed.

Fixation count, as a measure of attention loops and revisit behavior, was highest for Symbolic elements (13.03), followed by Source/Authority (12.50) and Text (11.34). The findings suggest that these AOIs were revisited several times by participants, especially when they occurred in emotionally rich or narrative highlighted areas of the ad. Most importantly, although textual AOIs had relatively large fixation durations, their TTFF was still significantly high at 366.21 ms, indicating that text was typically processed after visual and symbolic content. Such a visual-to-verbal route is also in line with previous descriptions of multimodal ad processing.

Image AOIs with faces or affectively charged movements also performed well across the board, with fast TTFF (232.56 ms) and relatively low fixation durations (246.57 ms). These images acted as attentional anchors in the way that they directed the eye of the observer to proximal message elements. They also drew fewer fixation counts, indicating early effective processing that did not need so many return visits—particularly where emotional significance was so obvious.

At the item AOI level, some of the items were observed as attention hotspots. Specifically, the headline text of Advertisement 1 (Ad1_HeadText) registered the highest fixations (27.59), whereas the breast icon of Advertisement 4 (Ad4_Icon(breast)) registered high engagement (Fixation Count = 29.55), as well as a longer duration of view. The government affiliation AOI (Ad6_Kivernisi) registered an unusually long fixation duration (743.23 ms), which indicated high engagement, most likely due to its proximity to empowering imagery or the familiarity of context.

These share a multicomponent view of visual persuasion: fast processing of visually prominent features such as websites or logos, followed by thorough processing of affectively or symbolically significant areas, and then a scrutinizing selection of textual information. Source cues and logos are likely to be identified initially but are most likely to be skirted in the sense of interpretation priority unless attached visually or semantically to focal persuasive features.

#### Further Visualizations

**Figure 4:**
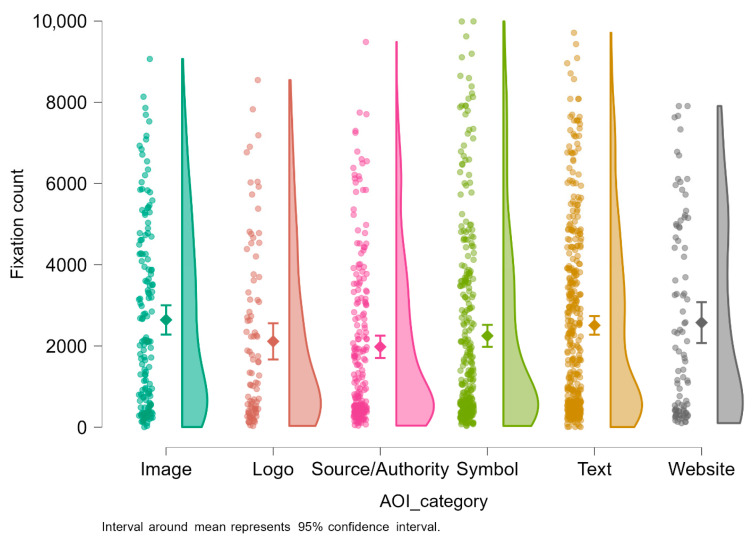
Distribution of fixation count across AOI categories. Raincloud plot depicting the distribution, density, and mean fixation count (with 95% CI intervals) across six area-of-interest (AOI) categories: Image, Logo, Source/Authority, Symbol, Text, and Website. Each dot represents a participant–AOI observation. The density plots illustrate the distribution shape, while diamonds mark the mean fixation count per AOI category.

To further explore the dataset, a number of visualizations were established for an in-depth understanding. Figure 4 shows fixation count distributions in six area-of-interest (AOI) categories for breast cancer awareness advertisements: Image, Logo, Source/Authority, Symbol, Text, and Website. Each participant–AOI observation is represented by one dot, with density distributions showing variation and the diamond markers showing the mean fixation count with 95% confidence intervals.

The results indicate that textual AOIs receive the largest average fixations, which aligns with readers viewing written health messages for longer periods of time. This indicates that content within text—headlines or emotionally framed calls to action—had some impact on drawing attention from the audience. Image AOIs, typically human subjects or emotionally evocative imagery, also received large fixation values, which aligns with their value in maintaining visual attention through emotional stimulation.

Symbol AOIs, like pink ribbons or icons of self-examination, had significant variations in fixations. While these symbols had a high focus, some may have been excluded due to design location or recognizability. Logo and Source/Authority AOIs (i.e., institution logos or titles) always elicited fewer fixations, suggesting peripherality in the viewer’s scan path. Finally, Website AOIs, action prompts, or campaign URLs had the lowest fixation counts, suggesting a low short-term interest in these targets.

**Figure 5:**
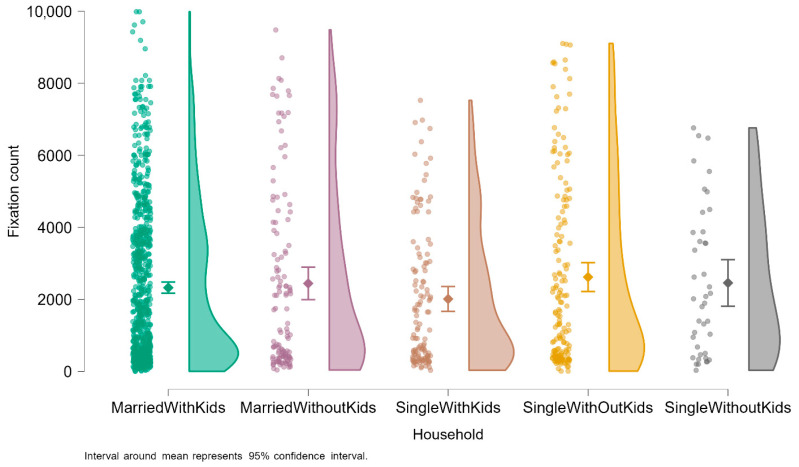
Raincloud plot depicting fixation count distributions across household types. Participants who were married with children exhibited significantly higher fixation counts than single participants with or without children. Violin plots display the density of values; diamonds represent group means with 95% confidence intervals.

In addition, Figure 5 shows the fixation count by family type from the breast cancer awareness eye-tracking study. Single points, boxplots of range and central tendency, and violin plots are plotted in each plot to see the underlying distribution.

Married with Kids was the most extreme group in terms of mean fixation counts, and it had the most extreme range with the highest values, indicating more extreme and heterogeneous visual attention. By contrast, Single with Kids and Single without Kids subjects had significantly lower fixation levels with more restricted distributions that imply a more consistent, contained pattern of eye movement. Also implied by the visual inspection is a trend for decreasing attention across household groups: from Married with Kids to Single without Kids.

**Figure 6:**
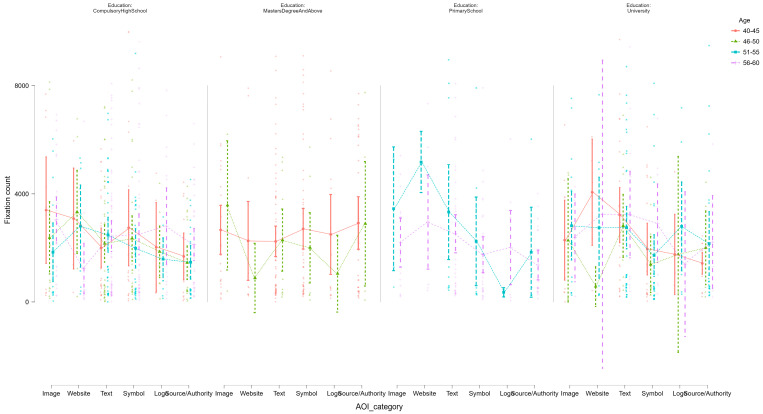
Fixation count across areas of interest (AOIs) by education level and age group. Each panel represents one education group (Compulsory High School, Primary School, University, and Master’s Degree and Above), with colored lines indicating mean fixation counts for age groups 40–45 (red), 46–50 (green), 51–55 (blue), and 56–60 (purple). Error bars represent 95% confidence intervals. Younger age groups, especially those with lower education, exhibited higher and more variable fixation counts, particularly on Image, Text, and Symbol AOIs. Higher-educated participants demonstrated more stable and evenly distributed attention across AOIs.

Figure 6 is a close-up of fixation count fluctuation by different areas of interest (AOIs) in breast cancer awareness advertisements according to level of education and then age group. There is a subplot for every unique educational category—Compulsory High School, Primary School, University, and Master’s Degree and Above—whereas colored lines are mean fixation counts by AOIs for 40–45, 46–50, 51–55, and 56–60 age groups.

In the 40–45-year-old participant group within the Compulsory High School group, these players had the largest and most variable fixations, especially on Image, Text, and Symbol AOIs. This may indicate higher visual interest or processing needed for emotional and informationally dense content in this sub-group. Older participants in the same schooling group (e.g., 56–60) produced flat profiles with lower overall average fixations, potentially indicating cognitive filtering or lower ad engagement. Participants with a master’s degree or higher showed more stable fixation patterns inside AOIs: stable but intermediate attention independent of content category. Here, the 40–45 group reflected the highest total fixation rate, though with less variance, reflecting more consistent scanning behavior. For the cohort at Primary School age, the 51–55-year-old group showed extremely strong fixation responses to Website and Image AOIs, possibly due to interest in visual and external call-to-action stimuli. Fixation lines of the oldest age group (56–60) are once more flat and symmetrically distributed, reflecting a passive viewing style. Lastly, among those who are university-educated, fixation trends converge by age but exhibit some peaks in Image and Source/Authority AOIs, particularly within the youngest and oldest age groups. Interestingly, this is the group with the smallest spread of AOIs and may be indicative of effective or schema-based visual processing.

Overall, this visualization uncovers intricate interactions between education levels and age in determining visual attention patterns. Younger and lower-educated participants are more likely to exhibit stronger and more diverse fixation behaviors, especially with respect to affectively relevant or informative ad items. In contrast, higher-educated participants engage in more effective and consistent scanning among ad items, perhaps because they can decode ads better or practice strategic selective attention.

### 4.2. RQ1—Early Capture and First-Hit Distributions

**Figure 7:**
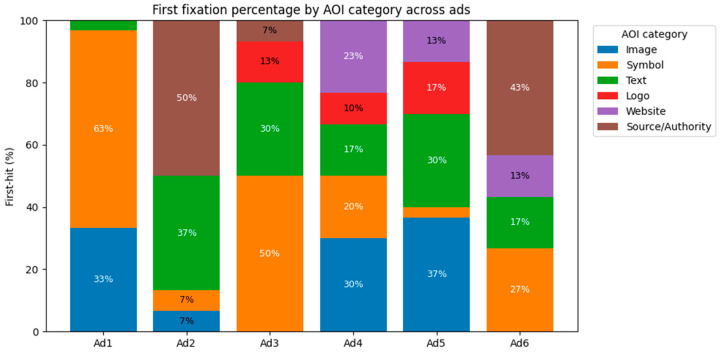
First fixation percentage by AOI category across ads (Ad1–Ad6). Stacked bars display the share of participants (N = 30) whose first fixation (minimum TTFF within a 10 s exposure) landed in each AOI category for each advertisement. Stacks sum to 100% within the ad. Categories shown: Image, Symbol, Text, Logo, Website, and Source/Authority. Numeric labels indicate within-ad percentages (labels suppressed for very small segments). Categories absent from a creative are omitted.

Across the creatives, visual features are more likely to draw attention first: Symbols prevail in Ad1 (63.3%) and Ad3 (50.0%), and Images capture Ad4 (30.0%) and Ad5 (36.7%). Message- and source-focused designs redirect early attention towards Text and Source/Authority—interestingly in Ad2 (Text 36.7%, Source/Authority 50.0%) and Ad6 (Source/Authority 43.3%). This trend suggests that early attention depends on inventive weighing: Salient individuals/things require initial attention, while highlighted headlines or institutional signifiers require first hits to message/source items (Figure 7). This is also consistent with our emphasis on design-ready diagnostics for early capture and assists in introducing the following analyses of prolonged attention (dwell dominance).

Overall, median TTFF measurements create “early attractors”. Subclasses that were classified as early capture had short TTFFs within the same advertisement (e.g., Ad1 Image = 29 ms; Ad5 Symbol = 29 ms; Ad4 Website = 18 ms; Ad3 Logo = 18 ms). Surprisingly, however, a fast TTFF did not always correlate with the highest first-hit share. For example, in Ad3, Logo had the quickest median TTFF (18 ms) but was responsible for only 13.3% first hits, while Symbols (50.0% first hits) were marginally slower (30 ms). These distinctions are proof that placement location and competition can create fast initial access to an item without it being the most typical first landing location. Table 4 presents first-hit percentages and median TTFFs by category and ad.

Collectively, the distributions indicate that early capture is heavily reliant on creative design. By placing focal imagery or high-salience symbols in salient, central, or high-contrast positions, they are likely to be fixated first (e.g., Symbols in Ad1: 63.3%; Symbols in Ad3: 50.0%; Images in Ad4: 30.0% and Ad5: 36.7%). In contrast, designs that highlight the headline or institutional cues direct initial attention to Text and Source/Authority (e.g., Ad2: Text 36.7%, Source/Authority 50.0%; Ad6: Source/Authority 43.3%). This is in line with the idea that viewers are sampling whatever attribute is most visually prominent or semantically primed by the design.

Median TTFF lengths overall provide evidence for the first-hit account: Categories that prevail in early capture also tend to have comparatively brief latencies to first fixation (e.g., Ad1 Image = 29 ms; Ad5 Image/Symbol = 31/29 ms; Ad4 Website = 18 ms; Ad3 Logo = 18 ms). There are, nonetheless, some diagnostic exceptions that demonstrate why TTFF and first-hit share ought to be interpreted together. In Ad3, Logo is fixated quickly (18 ms) but only represents 13.3% of initial hits, suggesting that while the logo is easily captured when it is targeted, it is not the favored landing point; rival elements (statistics icons and headline) draw the initial saccade. Likewise, high TTFF values for some categories (e.g., Ad4 Image = 2906 ms; Ad5 Website = 1187 ms; Ad6 Symbol = 3565 ms) reflect late access—presumably because of peripheral position, reduced contrast, smaller AOI size, or crowding from proximal attractors.

In practice, a fast TTFF depends upon paying attention in the first place; a class might be fast when selected but it is seldom selected first when other areas prevail in the initial struggle to draw attention (Ad3 Logo). Two, the early capture rank order is ad-specific and a question of the relative prevalence and positioning of competing items rather than some inbuilt superiority of a category. These diagnostics feed into the subsequent analysis of sustained attention (dwell dominance), where we look at whether the initial capture of the first glance also leads to retention and how such patterns differ by segment.

### 4.3. RQ2—Sustained Attention and Dwell Dominance

We measured sustained attention by constructing, for each ad, a tie-aware pairwise matrix *P* of areas of interest (AOIs), where $P_{ij}=Pr\left({\mathrm{dwell}}_{i}>{\mathrm{dwell}}_{j}\right)$ across participants viewing both AOIs (ties count 0.5). By construction $P+P^{⊤}=1$ and $\mathrm{diag}\left(P\right)=0.5$, these conditions held for all ads. For interpretability, every AOI had a dominance score of $S_{i}=\left(\bar{P_{i}}-0.5\right)\times 2\;\in \;\left[-1,\;1\right]$, with higher values indicating more frequent “wins” in within-ad dwell comparisons. We masked matrix cells backed by <5 participants in the figures.

#### 4.3.1. AOI-Level Dwell Dominance (Per Ad)

Across creatives, longer dwell was spent on message-carrying elements (headlines/text blocks and source or authority indicators) than on purely aesthetic images, except for advertising-related exceptions. Table 5 presents the top-three and bottom-three AOIs per ad by the S dwell-dominance measure. In Ad1, the face of the woman (Image) dwelled the most (S = 0.20), followed by the ribbon symbol (S = 0.08), whereas the headline and heart icon lost pairwise dwell (S = −0.13 and S = −0.14). In Ad2, the big text area (“Text”; S = 0.38) and heart symbol (S = 0.23) were most often winners, with political personality (Fofi Genimata; S = 0.13) also being positive; in contrast, the ornate “breasts” symbol (S = −0.35) and family photo (S = −0.21) were worst losers. In Ad3, the hospital portal label (S = 0.22) and logo (S = 0.20) were the strongest, with the overall headline being slightly positive (S = 0.12); the ribbon (S = −0.15) and a short text block (S = −0.22) were the weakest. In Ad4, the large block of text dominated (S = 0.20), followed by the sketch picture (S = 0.08); site band (S = −0.13) and headline (S = −0.16) performed poorly. In Ad5, the magnifying glass picture (S = 0.11) and middle text (S = 0.08) led the pack, while logo (S = −0.08) and headline (S = −0.07) lost. In Ad6, government/source (“Kivernisi”; S = 0.22) and site strip (S = 0.17) led the pack, with political figure also being positive (S = 0.12); the ribbon (S = −0.16), headline (S = −0.13), and fist picture (S = −0.13) lost. As an index, AOI dominance scores were positively correlated with the median dwell per AOI, i.e., for Ad3, there was a moderate to strong correlation between Si and median fixation duration (Pearson r = 0.69, Spearman ρ = 0.81), suggesting that the pairwise index agrees with the traditional dwell summaries but provides comparative, design-oriented information.

#### 4.3.2. Category-Level Dwell Dominance

Pairwise comparisons at the category level (Table 6) showed ordered patterns at the design layer. Text and Source/Authority were pair-wise consistent victors, whereas Image and Website were usually relegated to pairwise dwell losses in spite of occasional wins in first fixations. In particular, Source/Authority won in Ad2 (S = 0.54) and Ad6 (S0 = 0.61); Text won in Ad3 (S = 0.52), Ad4 (S = 0.41), and Ad5 (S = 0.72); Symbol won only in Ad1 (S = 0.56). Images fared the poorest in Ad2 (−0.65) and Ad6 (−0.53). This indicates that, after initial capture, message-informing text and source trust indicators maintain fixation more consistently.

Sustained attention was directed to semantically central features—headlines/text bodies and institutional/source markers—instead of decorative or illustrative images. Importantly, image-dominant creatives at early capture were not necessarily salient in dwell (e.g., Ad2 and Ad6: image strongly negative at category level), highlighting the difference between capture and processing. Exceptions were ad-specific (Ad5 magnifier image was slightly positive; Ad6 website AOI was positive amidst category-level website under-performance), in line with local salience and competition among elements. Pairwise collectively provides design-ready diagnostics: if a CTA or source banner regularly loses “gaze contests” to adjacent imagery or icons, it is actionable to adjust contrast, position, or crowding.

**Figure 8:**
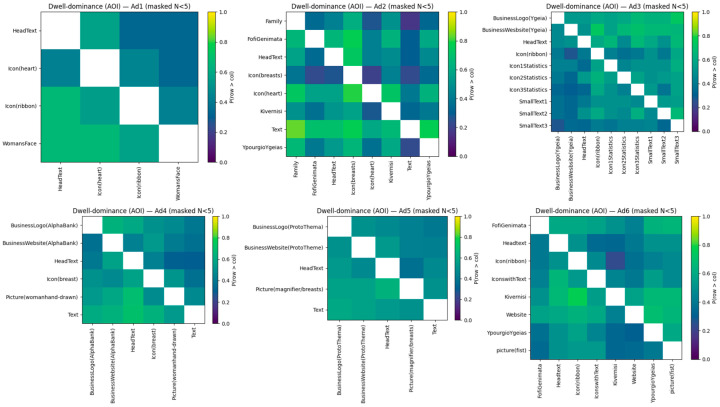
Pairwise dwell dominance (AOI level) for the six breast cancer screening ads. Each panel is a pairwise tie-aware matrix for a single ad. Cell values are the probability P (row AOI > column AOI in total fixation duration) across participants; ties receive 0.5, the diagonal receives 0.5, and support N < 5 participant pairs cells are masked. More considerable pairwise “wins” in sustained attention are indicated by warmer colors. Scan every matrix column by column compared to every row: rows loaded with lots of hot cells are dwell winners; columns loaded with lots of cold cells are dwell losers.

Through creatives, constant focus is placed on different elements depending on the design priority of the ad (Figure 8). For Ad1, Face of the Woman and the Ribbon icon perform better than HeadText and the Heart icon, indicating that portrait images and the screening ribbon hold attention longer than copy. Ad2 has a text-based structure: Body Text and Heart icon are victorious, along with Fofi Genimata (source cue), while Family photo and Icon(breasts) are defeated—message content and central symbol prevail over background image during prolonged processing. In infographic Ad3, BusinessWebsite(Ygeia) and BusinessLogo(Ygeia) won most pairwise battles, and HeadText was also preferred; small statistic blocks (e.g., SmallText3) and the Ribbon lost, implying that brand/source and the lead headline held people’s interest for longer than detailed data. For Ad4, the strongest dwell winners are the central Text block and Hand-drawn image; HeadText, BusinessWebsite(AlphaBank), and Icon(breast) lost, suggesting that the central copy panel and image are “stickier” than any individual brand/CTA element. For Ad5, the most wins among leads are the lead Picture (magnifier/breasts) and Text, and BusinessLogo(ProtoThema), HeadText, and the Website are relatively weaker—again suggesting the superiority of the central image and body copy. Finally, Ad6 is policy-framed: Website and Kivernisi (government identifier) are the strongest dwell winners, followed by the positive Fofi Genimata; Icon(ribbon), Headtext, and picture(fist) are the standard losers, and YpourgioYgeias is weakly negative. Overall, the matrices answer RQ2 by determining, per ad, which items usually “win” gaze competition and thus are most to blame for maintaining processing. These pairwise probabilities augment the first-hit outcomes by illustrating that early capture is not necessarily equivalent to dwell dominance (i.e., small logos or quickly encountered icons can still be overtaken by sustained attention when pitted against central text blocks, strong images, or prominent source cues).

### 4.4. RQ3—Subgroup Attention Patterns and Audience Shifts in Early Capture

We looked for variations in early attention (the probability an AOI category was first looked at; “first-hit %”) between demographic groups. First-hit percentages by each ad and by Age, Household, and Education are displayed in Table 7, Table 8 and Table 9. For ease of presentation, we also calculated the delta heatmaps of each subgroup’s first-hit distribution minus the within-ad overall distribution. Given that this analysis is exploratory and some cells are small, we present the direction and not the exact magnitude.

Three general trends were observed between commercials. First, source/authority cues in ads attracted earlier fixations in older and lower-educated groups (e.g., Ad2 and Ad6), whereas text attracted earlier attention in more highly educated groups (e.g., Ad2, Ad5). Second, symbolic/iconic components attracted early attention within most groups (e.g., Ad3 for age group 51–60 and for one-person households) frequently prior to logos and images. Third, family structure was isomorphic to where eyes first fell: Married viewers started more frequently with source/authority (Ad2 and Ad6), while single viewers started with text (Ad2 and Ad5) or symbols (Ad1, Ad3, and Ad6). These subgroup tendencies add dwell-dominance results by indicating where viewers started before longer viewing.

When older viewers were shown prominent source cues, older viewers had a higher likelihood of starting on Source/Authority (Table 7). For instance, in Ad2 and Ad6, the 56–60 group exhibited a higher percentage of first fixations to Source/Authority than the study mean (for instance, Ad2: 56–60 = 66.7% vs. 40–45 = 44.4%). Younger viewers, on the other hand, were more likely to start on Symbols/Images (for instance, Ad1 ribbon; Ad5 magnifier). In Ad3, initial contacts shifted from Text between 40 and 45 years (44.4%) to Symbols for later groups (≈55–71%).

Household structure also aligned with where individuals first looked (Table 8). Married respondents would start on Source/Authority when government/organization cues were present (e.g., Ad2: 66.7% married no children; Ad6: 52.6% married with children). Childless respondents favored starting on Text in copy-forward structures (Ad2 and Ad5: 100% on “single without children”) or Symbols when prominent (Ad1, Ad3, and Ad6).

There was an obvious content-type gradient (Table 9). More educated consumers were more likely to start on Text (e.g., Ad2 University = 57.1%; Ad6 Masters+ = 50.0%; also Ad5 University = 42.9%), while compulsory/secondary consumers were more likely on Source/Authority (Ad2 = 69.2%; Ad6 Primary = 100%) or on Symbols/Images if these were competing with copy (e.g., Ad1 compulsory/HS Symbols = 53.8%). In Ad3, University participants heavily preferred Symbols (85.7%) over Text for first fixations.

Together, the group contrasts reveal first-fixation patterns to be design-fixed but audience-variable: Authority-leading styles address older and family audiences first; data-heavy or copy-leading styles address higher-education audiences; strong symbol or image content preferentially addresses younger and single viewers. These patterns complement dwell-dominance findings by revealing where audiences first engaged prior to extended watching and must be considered hypothesis-generating patterns to be tested in larger samples.

**Figure 9:**
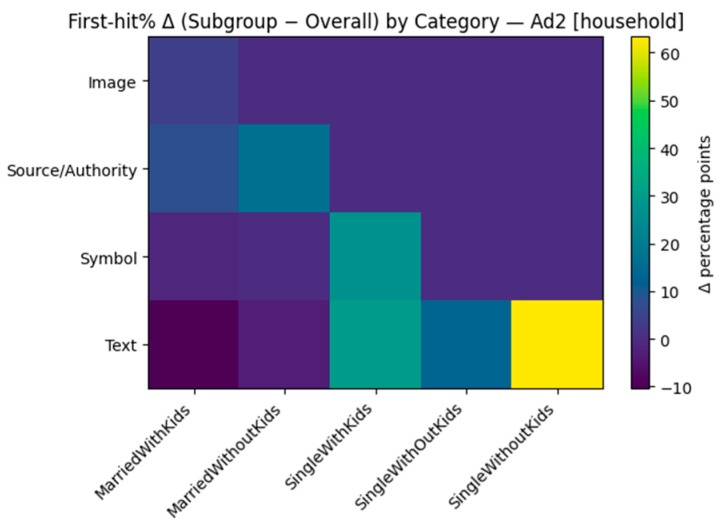
Example of first-fixation shifts by household (Δ subgroup − overall)—Ad2. Cells show percentage-point differences between each household subgroup and the overall sample within the ad and AOI category. Warmer colors indicate categories that captured a greater share of first fixations for the subgroup than overall; cooler colors indicate a smaller share. Columns are household strata; rows are AOI categories.

**Figure 10:**
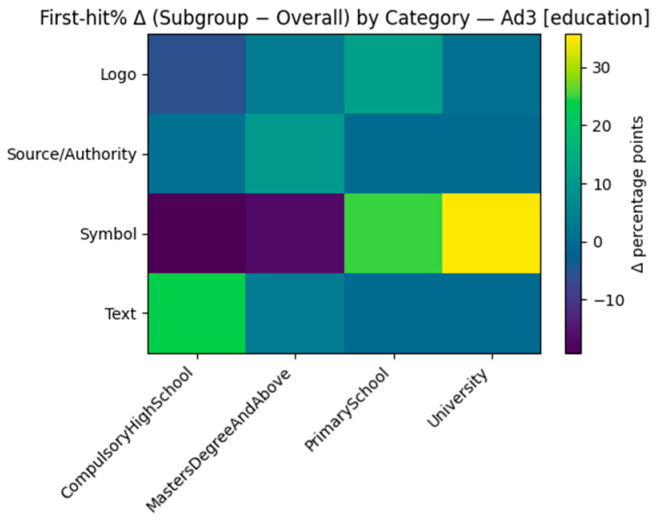
Example of first-fixation shifts by education (Δ subgroup − overall)—Ad3. Same encoding as Figure 8. Rows list AOI categories used in Ad3 (Logo, Source/Authority, Symbol, and Text).

Zooming in, the family deltas track those with general tendencies but diverge by creativity. In Ad1, singles with no children are over-indexed on Symbols (ribbon/heart), and those married with kids lean slightly towards Image; Text never over-indexes. Ad2 shows a copy-forward entry: Singles with no kids all move strongly to Text, and married with kids move towards Source/Authority; images are under-indexed across families. In Ad3, single persons without children prefer Symbols, while single persons with children trend toward Text/Logo (with Source/Authority close to baseline). Ad4 reverts to pictures—single persons without children have a large positive Δ for hand-drawn Image, while married persons without children prefer Text; Website is never first. Ad5 once more indicates that childless singles prefer Text, and single persons with children prefer Image (with Logo/Website rarely first). Ad6 breaks down tidily: Singles with no children over-index on Symbols, singles with children on Website, and married with no children on Text, and Source/Authority is over-indexed with respect to couples with family. Examining education next, Ad1 has Primary and Masters+ audiences over-indexing on Symbols and University leaning towards Image (with Text typically low); Ad2 has Compulsory/HS and Primary inclining towards Source/Authority, compared to University/Masters+ inclining towards Text (Primary also leans slightly towardsImage). Ad3 has a distinct gradient—University (and Primary) to Symbols, Compulsory/HS to Text, and Logo positive at the tertiary level. In Ad4, Primary over-weights Logo/Image, University skews towards Text, and Website hardly receives the first fixation. Below, we provide illustrative examples of first-fixation shifts by household (Δ subgroup − overall) for Ad2 (Figure 9) and first-fixation shifts by education (Δ subgroup − overall) for Ad3 (Figure 10).

#### Early vs. Sticky (Entry vs. Dwell)

We compared early capture and prolonged processing for every ad to a scatter plot with the *x*-axis as the first-hit percentage (chance of being the earliest fixated in an ad) and the *y*-axis as its dwell-dominance score. S ∈ [−1, 1] is our pair-wise, tie-aware measure of how frequently an AOI defeats other AOIs “dwell duels” within the same ad. A reference vertical line is the within-ad median first-hit %, and a reference horizontal line is S = 0. Together, they divide AOIs into four design-meaningful quadrants: early and sticky (upper right), early but not sticky (lower right), late but sticky (upper left), and neither (lower left).

**Figure 11:**
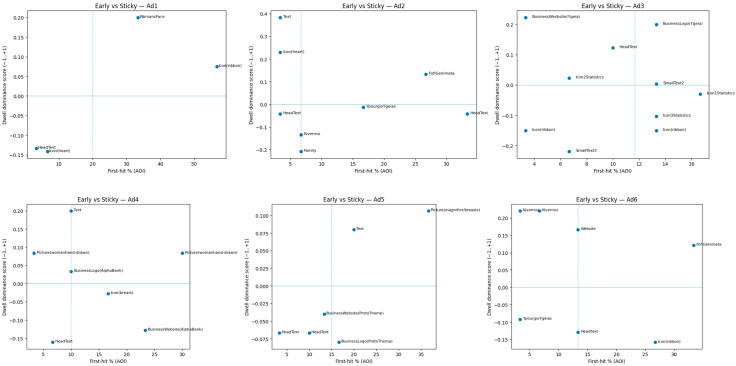
Early vs. sticky attention (per ad). Each point is an AOI (labels show AOI names). The vertical line is the within-ad median of first-hit %; the horizontal line is S = 0. **Top-right** AOIs both capture the first fixation and hold gaze; **top-left** AOIs arrive later but keep attention; **bottom-right** AOIs capture early but do not retain attention; bottom-left AOIs do neither.

Across ads, trends were consistent and design-fitting: In Ad1, the ribbon is early and sticky, the face of woman is late but sticky (firm pull once located), and headline and heart are neither; in Ad2, body copy is sticky but late, headline is early but not sticky, Fofi Genimata is early and sticky, and family photo and government seal lose with respect to dwell; in Ad3, logo is early and sticky, the hospital website tag is sticky but late, and ribbon with some of the little blocks of text underperforms; in Ad4, the hand-drawn photo is early and sticky, the strip on the website is early but not sticky (capture without sustaining interest), the leading block of text is late but sticky, and headline underperforms; in Ad5, the magnifier picture is early and sticky, the center text is sticky at about mid-early time, and logo and website are not sticky; in Ad6, the political endorser (Fofi Genimata) is early and sticky, the government label and website are sticky but late, the ribbon is early but not sticky, and headline is not sticky (Figure 11).

The composite entry-dwell view explains why some things “work.” Elements linked to authority (Ad2 and Ad6) are able to capture and maintain attention, particularly from older readers, but typographic highlighting may be necessary for headlines to maintain dwell after the initial fixation capture. Data- or copy-heavy designs (Ad3) always anchor attention but can benefit from greater contrast or proximity to probable entry points to be noticed sooner (e.g., move logo/URL into first path). Practically, move late-but-sticky AOIs farther up visual entryways (e.g., Ad3 website/logo; Ad4 copy) and clear clutter surrounding the early-but-not-sticky elements (e.g., Ad4 website band; Ad5 headline) so that early capture equates to useful seeing (Table 10).

### 4.5. Sanity Checks and Robustness

We conducted two robustness checks to ensure that the pairwise indices used throughout the paper behave as intended. We first checked the two algebraic properties that derive from our pairwise construction: (a) symmetry around 0.5 of the probability matrix, i.e., $P+P^{⊤}=1$ off-diagonal, and (b) neutrality on the diagonal, i.e., $P_{ii}=0.5$. Both properties held exactly (numerical tolerance) for the AOI-level dwell-dominance matrices and for the category-level TTFF precedence matrices and for each ad when tested ad by ad (Table 11). As a robustness convention, we suppressed any cell supported by fewer than five overlapping observers when showing matrices; all calculations preserved ties as 0.5. Second, as a robustness check, we tested whether AOIs that are high on the dominance index also receive greater dwell when reading conventional summaries. For every advertisement, we correlated the AOI-level dominance score with the AOI’s median fixation duration (ms) of the benchmark tables. Correlations were positive and moderate-to-strong for all ads (Pearson r range = 0.68–0.98; Spearman ρ range = 0.50–1.00; Table 12), suggesting that dominance ranking is consistent with traditional dwell measures while maintaining a comparative, design-ready significance.

Together, these robustness checks confirm that (a) the pairwise matrices are internally consistent by construction and (b) the rankings produced represent real differences in depth of processing rather than being artifacts of the scoring system.

## 5. Discussion

### 5.1. RQ1—Early Capture (First Hit/TTFF)

Across the creatives, initial focus was strongly design-driven. Faces, pictorials, and icons most typically drew first fixation in symbol- or image-dense designs (e.g., Symbols in Ad1 = 63.3%, Symbols in Ad3 = 50.0%, Images in Ad4 = 30.0% and Ad5 = 36.7%). Message- and source-directed designs redirected entry to Text and Source/Authority (Ad2: Text = 36.7%, Source/Authority = 50.0%; Ad6: Source/Authority = 43.3%). Median TTFFs on return precisely reflected these distributions, with brief latencies for gateway categories (e.g., Ad3 Logo = 18 ms; Ad4 Website = 18 ms; Ad5 Symbol/Image = 29–31 ms). Interestingly, there were also some diagnostic dissociations: Ad3’s logo, with access being extremely rapid (18 ms), had only 13.3% first hits, showing that accessibility of an element does not necessarily promise it to be the modal entry point if other attractors direct the initial saccade elsewhere. Similarly, big TTFFs in certain groups (e.g., Ad4 Image = 2906 ms; Ad5 Website = 1187 ms; Ad6 Symbol = 3565 ms) indicate slow access owing to peripheral location, low contrast, small AOI size, or local crowding. Generally, within-ad first-hit distributions were limited to one or two categories (most often one third to two thirds of viewers), stressing that the limited range of design choices highly predicts where attention starts.

They align with well-established results that an image is scanpath-prioritized over text when the image is visually salient, and this aligns with the Limited Capacity Model (LC4MP): First, limited processing capacity is devoted to the most salient objects [5,12,27]. In parallel, our Ad2 and Ad6 findings demonstrate that the use of institutional or argumentative markers can direct first fixation onto Text and Source/Authority, which aligns with ELM/EPPM accounts in which centrally diagnostic markers capture attention when made visually salient [1,11,15]. The first-hit benefit of pictures and symbols likewise transfers to public-health warning research, where pictorials all consistently capture earliest fixations and high-density copy consistently needs salience boosts simply to be viewed at all; our “source-forward” designs, on the other hand, show how design can overcome that text-based default weakness by boosting contrast, size, and location [3,15,25].

Two implications for practice follow. First, “fast” TTFF is a function of choice: a category can be rapidly capturable when cued (Ad3 Logo), but never in a million years will it serve as the portal of entry for the population if the surrounding areas control pre-attentive competition. Second, the rank order of capture early on is ad-specific and an outcome of relative salience and spatial competition and not due to any absolute superiority of a category. As such, design diagnostics reveal the existing gateway items by ad and near-misses in need of contrast, proximity to likely approach paths, or diminished local competition to gain the first glance. They point RQ2’s inquiry of whether winners of the first hit also maintain attention (dwell dominance) and pose RQ3’s challenge of whether gateways vary across audience segments.

### 5.2. RQ2—Sustained Attention (Dwell Dominance)

Pairwise dwell-dominance matrices and AOI rankings confirm that, when fixated, message-delivering elements, headline/body copy, and source/authority markers more consistently maintain the gaze than ornamentation imagery, with ad-typical exceptions. On the AOI level, Ad1 favored the woman’s face and ribbon (S ≈ 0.20, 0.08) over headline and heart (S ≈ −0.13/−0.14). Ad2 was overwhelmingly copy-dominated (Text S ≈ 0.38) with a supporting symbol (heart, S ≈ 0.23); family photo and stylized breasts icon fell out of dwell (S ≈ −0.21/−0.35). In infographic Ad3, tag website and logo reigned (S ≈ 0.22/0.20) with principal headline being positive and small statistic blocks and ribbon underperforming. For Ad4, long texts and hand-drawn figures were the winners (S ≈ 0.20/0.08), magnifier image and mid-text in Ad5 dominated (S ≈ 0.11/0.08), and Ad6 was source-dominated, with the government label and website leading (S ≈ 0.22/0.17). Category-level summaries agree on the same trend: Text and Source/Authority most frequently “win” pair-wise comparisons (e.g., Ad2–Ad6), while Image loses even when it won first fixation (precisely Ad2 and Ad6). These findings suggest that in initial capture, semantically central items are more likely to be given prolonged processing time tagged with encoding [7,8,35,49].

This is in accordance with previous results that longer fixations and revisits, and not first glance, are more memory diagnostic and more convincing. In advertising, attention to brand mark and text predicts memory for the brand; in health warnings, longer dwell time on warning messages enhances recall when salience is high [25,35,50]. In LC4MP, prolonged fixation signals resource investment in encoding and memory; our matrices decide on which objects receive that resource in competitive setups. The salience of Text and Source/Authority in Ad2, Ad4, Ad5, and Ad6 is also consistent with ELM/EPPM: If arguments or reputable endorsers are visually highlighted, respondents process message content centrally, and representative images (particularly background or decorative) attract less sustained processing [15,16,25,51]. The semantically interesting exceptions in our findings are content-diagnostic visuals, including Ad5’s magnifying glass, a salient cue to screening, for which its dwell gain illustrates that tightly connected images with respect to the call to action can act like arguments rather than lures.

Two tests of validity underpin these inferences. First, all pairwise matrices met construction-implied axioms (off-diagonal: P + Pᵀ = 1; diagonal = 0.5) at AOI and category levels and for each ad (§5.5), thus ensuring internal consistency. Second, AOI dominance scores were moderately to strongly and positively correlated with benchmark median dwell across ads (Pearson r ≈ 0.68–0.98; Spearman ρ ≈ 0.50–1.00), as the comparative index not only follows standard dwell summaries but also provides a design-ready, head-to-head interpretation. Together, these controls validate the conclusion that the identified dwell winners truly do receive additional processing.

In practice, the early-vs.-sticky plots translate the matrices into edits. “Late-but-sticky” items (e.g., Ad3 website/logo; Ad4 long copy) are best positioned for repositioning and contrast enhancement close to natural entry points so that their processing benefit can be ignited earlier [3,33]. “Early-but-not-sticky” items (e.g., Ad4 website band; some headlines) need simplification, typographic support, or semantic linking to turn capture into processing. Where authority badges attract dwell (Ad2 and Ad6), keeping the CTA/URL in their view can leverage that attention instead of deflecting it elsewhere [51,52]. Where decorative images usually lose dwell, lowering their salience or adding short, high-value micro-copy can limit competition and boost informational throughput [9,13]. In short, the dwell-dominance framework decides what is worthy of protection, promotion, or redesign, converting public health practice recommendations from decades past (e.g., prioritize CTAs and reliable sources, restrict visual competition) into concrete, ad-specific data.

### 5.3. RQ3—Audience Shifts (Age, Household, and Education)

Subgroup breaks of first-hit% (Table 7, Table 8 and Table 9) and Δ heatmaps (subgroup − overall; Figure 9 and Figure 10 reveal systematic, theory-consistent variations in early capture. In conditions where source cues were prominent, older viewers placed a greater proportion of first fixations on Source/Authority (e.g., Ad2, Ad6: 56–60 > 40–45), while younger viewers demonstrated greater initiation probabilities on Symbols/Images (e.g., the ribbon in Ad1; the magnifier in Ad5). Family structure corresponded naturally to entry points: Married participants, particularly child participants, would likely start on Source/Authority when present (Ad2 and Ad6), while childless participants would start on Text in copy-forward orientations (Ad2 and Ad5) or on Symbols when salient (Ad1, Ad3, and Ad6). Education had the strongest gradient: higher-education segments began most frequently on Text/Website (e.g., Ad2 University ≈ 57%; Ad6 Masters+ ≈ 50%), whereas compulsory/secondary or primary-school segments began on Source/Authority or Symbols/Images whenever these conflicted with copy (e.g., Ad2 Compulsory/HS ≈ 69% Source/Authority; Ad1 Compulsory/HS ≈ 54% Symbols).

These trends align with LC4MP: Restricted processing capacity is initially devoted to cues that are schematically pre-activated or highly salient [16,26]. Salience works differently in our findings across strata—pictorial and symbolic cues are the preattentive attractors for younger/single audiences, while institutional badges are salient anchors for older/family audiences. They also correspond to ELM/EPPM accounts: when centrally diagnostic content is visually salient and the audience is able/motivated (proxied by education), copy/statistics appeal to consumers; when motivation/ability is weaker, or when peripheral cues are particularly salient, imagery and endorsement drive purchase decisions. This aligns with health-warning and advertising materials demonstrating that pictorials override prior fixations in younger audiences, but text and source/brand attention are more effective at predicting future recall when the viewer processes centrally [11,16].

Supplemental age × education line plots of mean fixation counts throughout our atlas replicate these patterns: Younger viewers from lower schooling strata had higher, more variable fixation counts on AOIs, as theorized for exploratory scanning; master’s-level viewers had even, schema-like distributions, indicating strategic selective attention in favor of message-carrying regions once found [6,16]. Combined with the early-vs.-sticky quadrants, these subgroup findings inform who looks where first, and what then maintains looking—a convenient pair for segmentation.

Design consequences are immediate. For family or mature demographics, control-framed configurations (endorser/seal + CTA proximity) will likely be most effective at drawing entry and must position the CTA/URL in the same foveal neighborhood to turn initial capture into dwell. For educational purposes, copy density or data-driven structures might work, but they must ensure typographic differentiation and proximity to probable entry portals (icons and faces) to avoid leading to delayed discovery [3,17]. For youth and single audiences, image or symbolic strong elements can be used as deliberate portals if they are placed spatially in conjunction (nesting, arrows, and proximity) with eligibility or CTA words so that the risk of overpowering imagery hijacking attention from action signals is reduced.

Lastly, we note the exploratory nature of such subgroup differences and limited cell sizes within certain strata; interpretations serve to caution against direction vs. particular magnitude. Age/Household/Education deltas, though, provide hypothesis-generating evidence to guide audience-specific micro-edits: authority signals to stimulate older/family audiences; copy contrast and positioning for high-education audiences; and symbol-to-CTA complementarity for young/singles audiences [3,33]. These patterns fill out the dwell-dominance results by showing where and how viewers begin before processing becomes extended, guiding both creative refinement and directed media delivery.

To transform patterning of early capture and long-term residence into practical guidance, we abstracted the AOI benchmark tables, early-vs.-sticky quadrants, and category-level transition diagrams into brief design heuristics (Table 13) and per-ad action guidelines (Table 14). Three patterns are seen across creatives: (a) CTA text is not typically an initial focus of attention compared to prominent imagery; it is late but persistent, proposing a benefit in enhanced typographic contrast and the positioning of copy near the prevailing entry cue; (b) iconic/symbolic content tends to dominate text for entry, and this can be optimized by nesting or pointing icons at the CTA; and (c) logos/website labels tend to be persistent but found late, proposing proximity or contrast modification to the natural entry flow. In control-dominant designs, source/endorser marks may become early-and-sticky and powerful but with the penalty of drawing attention away from neighboring calls to action unless micro-contrast and spacing are controlled. These heuristics are derived from relative dominance patterns and the first-pass flux demonstrated here and are meant to guide iterative creative optimization, not population-level causal inference.

### 5.4. Implications for Theory

Our findings refine a dual-process model of PSA attention. The first-hit/TTFF phase explains preattentive capture by visual saliency, and location, faces, icons, and centrally placed badges win the initial competition when they are highly visible visually, while the dwell-dominance stage follows continuous, capacity-exercising processing that is closer to understanding and potential persuasion. This differentiation is in accordance with LC4MP, according to which attention is a limited resource allocated first to the salient and then to what will assist encoding and storage, and ELM/EPPM, which both make predictions that centrally diagnostic cues (e.g., efficacy text, credible source) need to draw on in order to elicit elaboration [6,26]. The dissociations that we are seeing—fast-TTFF items that are not entry points for modality and first items that cannot be fixated—illustrate the inadequacy of separate measures: bottom-up capture (first-hit/TTFF) and top-down assessment (dwell) are complementary activities, not redundant ones [4,18].

The pattern—images capturing entry while text and source cues control dwell—counters traditional ad research (pictorials lead text in scanpaths) but aligns with health communication scholarship that longer dwell is more predictive of recall than “what was seen first”. In dual-route language, dominant pictorials are peripheral gateways that require support from central information to move processing from detection to elaboration. Alternatively, copy- or authority-forward designs may circumvent peripheral capture and lead consumers directly to central cues, corresponding to circumstances where central routes are more popular (ability/motivation and diagnostic of cues) [7,24,28]. Our subgroup shifts (older/family on Source; higher-education on Text/Website; younger/single on Symbols/Images) generalize this theory to account for how viewer attributes influence which signals are preattentively emphasized (schema-guided selection for more able viewers; affective/imageric capture for others), connecting visual attention to capability and motivation constructs at the heart of ELM/EPPM.

Methodologically, the pairwise, tie-aware dominance approach gives a concise, assumptionless way of transforming raw gaze into relative probabilities in line with Bradley–Terry/Thurstone philosophy. The matrices satisfy the expected axioms (P + Pᵀ = 1; diag = 0.5), and the derived dominance ratings corresponding to standard dwell aggregates offer construct validity, as well as design-interpretable scales (who “wins” gaze and by how much). Along with the early-vs.-sticky state-space (x-entry, y-dwell dominance), this provides a two-dimensional theoretical perspective: AOIs can be described as peripheral magnets (early/not sticky), central anchors (late/sticky), workhorses (early/sticky), or bystanders (neither). Third, the atlas-style, subgroup-conscious visualizations respond to recent pleas for reproducible pipelines in eye-tracking: through routine-smoothing, AOI benchmarking, and low-N masking, they present a theory-consistent road from attentional competition to actionable hypotheses regarding message processing in PSAs.

## 6. Practical Implications

This research aimed to provide design-ready diagnostics through the decoupling of initial capture (first-hit %, TTFF) from ongoing processing (dwell dominance) and through the demonstration of how these patterns differ across audience strata. The findings are significant for specific policy, management, and pedagogical settings that routinely create or commission informational graphics.

### 6.1. For Policymakers and Government Agencies

Procurement briefs and guidelines need to formalize attention guardrails. Since Text and Source/Authority tend to capture sustained attention, whereas Images/Symbols capture the first glance, the requirements need to mandate the availability of a readable, high-contrast CTA/eligibility block co-located with a salient entry cue and source badges placed in close proximity to the CTA so credibility does not divert attention [4,18,25]. Pre-launch testing must ensure that CTAs eschew the “early-but-not-sticky” quadrant. The subgroup results corroborate unique segmentation: family and older households to Source/Authority, university groups to Text/Website, and young/single groups to Symbols/Images. Ordering variant creatives for priority segments is sensible while maintaining the integrity of the CTA–source combination [7,25]. Agencies may employ the atlas pipeline, heatmaps, first-hit distributions, early-vs.-sticky quadrants, and pairwise dominance matrices as an inexpensive go/no-go pretest prior to committing to media spend. The internal consistency of the matrices and their concordance with traditional dwell measures render them appropriate screening diagnostics (albeit not inferential proof). Lastly, designing for accessibility and equity is crucial: when copy drives ongoing processing, ensure readability and language access; when entry depends on imagery—particularly in lower-literacy environments—match it thoughtfully with the CTA to prevent attention from lingering on decorative aspects.

### 6.2. For Business and Campaign Managers

Translate learnings into a lean production checklist that resists over-indexing on what appears salient but is not. Shield early-and-sticky pieces from crowding; activate late-but-sticky pieces by placing them closer to the probable entry and strengthening typographic hierarchy; eliminate early-but-not-sticky pieces by removing competitors and inserting micro-copy; and revisit neither-nor items, adding load and minimal payoff. Carry out conversion design, not capture design: Since images and icons are likely to attract first clicks but not retain attention, pair visual magnets with direction cues (nest CTA in the image; employ iconography pointing to or enclosing the CTA) and put logos/URLs—frequently sticky but tardy—along the route from entrance to copy instead of out on the perimeter. Install attention KPIs by objectives: for awareness, achieve enough first-hit share to the message gateway (headline/source block); for action, measure the shift of CTA movements from late-but-sticky to early-and-sticky over iterations, using the dominance score as a comparative KPI aided by recall or click-through where possible [4,25]. Budget micro-edits, contrast, hierarchy, spacing, and proximity—instead of carrying out full overhauls—and test movement in the target quadrant with rapid A/Bs using atlas graphics prior to production.

### 6.3. For Educators and Health-Promotion Teams

Match processing style and scanning with the ad format. With professional or university audiences, highlight statistic panels and copy-forward designs; with family or older audiences, present great source/authority badges with the CTA to take advantage of initial trust; and with youth-oriented outreach, utilize symbols/images as hooks but place the CTA inside the visual for smoother transitions to texts. Place the most important message (eligibility, next step) in one or two saccades from the entry point, utilize good headings and micro-copy to reduce cognitive load, and employ redundant coding (icon + brief verb phrase) to compensate for varied literacy. Train front-line personnel to interpret attention diagnostics—the first-hit distribution, dwell matrix, and early-vs.-sticky panel—so they can brief in-house designers or vendors with clear, actionable briefs (e.g., “our CTA is late-but-sticky—move it earlier toward the entry image and boost contrast”).

### 6.4. Cross-Cutting Practices

Institutionalize a test–iterate loop: prototype to quantify (first-hit, dwell dominance), rebuild, and repush, with regular outcome tracking (recall, appointment rate) to verify whether attention shifts are aligned with action [16,17]. Standardize assets and templates that impose winning combinations (CTA + source proximity; icon-to-CTA mapping; headline length constraints) to drive maximum baseline quality with flexibility for variants for each audience. Lastly, record assumptions and constraints: The current findings are from six breast cancer screening creatives and a mid-sample and are hypothesis-generating; if extrapolating to other subjects or platforms (video and interactive), re-run the same diagnostics to verify whether the same factors remain early or sticky and be transparent about smoothing, AOI borders, and masking to ensure comparability.

## 7. Conclusions, Limitations, and Future Directions

This paper introduced a replicable, design-centered analytics pipeline that separates early capture (first-hit %, TTFF) from long-term processing (pairwise dwell dominance) and graphically visualizes both on AOI and category levels. For six breast cancer screening ad creatives, early attention was significantly design-dependent: Image/symbol-driven layouts drew first fixations to pictorials and icons, while message- and source-led layouts deflected entry to Text and Source/Authority. Longer-term focus, however, clustered around message-carrying objects—bundles of text and assertions of control—rather than around ornamented images. “Early-vs.-sticky” quadrant charts balanced these phases against each other, enumerating feasible instances (i.e., late-but-sticky CTAs and pages that are worth repositioning; early-but-not-sticky objects worth tidying up or micro-copying).

Subgroup strata (age, household, and education) created audience-sensitive entry paths: older/family households to Source/Authority, higher-education audiences to Text/Website, and younger/single audiences to Symbols/Images. Internal diagnostics validated the measures: All pairwise matrices met symmetry/diagonal axioms, and dominance scores were moderately to strongly linked with benchmark dwell summaries. As a whole, the atlas, matrices, and quadrants transform raw gaze into design-ready heuristics and are transparent and replicable.

This research was designed as a descriptive, design-to-decision pipeline; the same design decisions that make it fast and interpretable also imply explicit directions for extrapolating and scaling up the evidence base [16,17]. First of all, this research was constructed as a design-to-decision descriptive pipeline; the same decisions that make it readable and effective also have explicit prescriptions for scaling and external validation. The atlas initially addresses pattern discovery in a moderate cohort and six static creatives. A commensurate next step is a large-scale preregistered replication with a larger, stratified sample that allows for sharper estimates of subgroups (e.g., whether older audience members are more likely to initiate on Source/Authority than younger audience members). Multilevel models nesting AOIs in ads and participants—estimation through bootstrap or Bayesian estimators—can place uncertainty bands around dominance scores and subgroup deltas without sacrificing comparative, design-oriented interpretation [3]. Consistent with the atlas’s stance, we emphasize descriptive diagnostics here; to convey uncertainty, we will report nonparametric bootstrap CIs in an expanded appendix and, in the planned replication, conduct a priori power analyses and preregistered multilevel inference. Second, our interest here is static, print-like stimuli within a single cultural–linguistic context. Taking the pipeline to dynamic media (TV ads, social/video streams, and scrollytelling) will challenge whether the static entry–dwell dissociation generalizes when motion, pacing, edits, and interaction influence salience. This will need time-varying AOIs, shot-change processing, and time-normalized dwell/precedence metrics. Parallel replications across languages and health systems will untangle universal design regularities from context-specific conventions (e.g., typography and iconography) [17,26]. As our sample was recruited via university mailshots, clinics, and local networks, generalizability to broader populations is limited; the larger replication will diversify recruitment to mitigate sampling bias. Third, AOIs were delineated at a designer’s level of interest (CTA, headline, symbols, and sources). Next, research must rigorously test analysis decisions: perturb AOI boundaries; change fixation-detection thresholds and heatmap kernels; and experiment with alternative view geometries. Perturbation-stability of first-hit shares and pair-wise dominance reported as measures of robustness would guide best-practice defaults to practitioners. Though our pairwise method is naturally apt for within-ad measures, more general cross-ad inferences can be stabilized by having calibrated anchors: (a) possess a restricted set of standardized reference AOIs that occur across creatives (e.g., a templated CTA card) so as to enable the partial pooling of dominance parameters and (b) blend pairwise wins with common-timescale dwell so that creatives with varying AOI counts/sizes can nonetheless be contrasted on an interpretable composite [12,31].

In addition, although first hit and dwell are proximal attention metrics, campaigns ultimately aim to action and memory. Future research must tie movement in the early-vs.-sticky quadrants to later metrics, such as unaided/aided recall, site visits, and appointment settings, using field A/Bs and pre–post-deployments. In practice, one can iterate micro-edits (contrast, proximity, and decluttering), make a CTA move from late-but-sticky to early-and-sticky, and test corresponding gains in conversion metrics [12,13].

Also, segmentation can be expanded beyond age, household, and education to include equity and access predictors that are more directly relevant to public health: visual acuity, language ability, health literacy, and screening history. The same tests can establish whether suggested solutions (e.g., increased contrast, redundant icon and verb coding, increased CTA–source pairing) span attention gaps across lower-literacy groups without abbreviating engagement elsewhere. Finally, design causality has to be addressed through targeted experiments. Factorial manipulations in size, contrast, proximity, and spacing can probe dose–response relationships behind the heuristics (e.g., how much contrast lift is necessary for a headline to move from the “early-but-not-sticky” quadrant?). Since the pairwise matrices are symmetrically/neutrally aligned by definitions and coincide with the median dwell, they provide an effective first endpoint for such experiments, with secondary corroboration arising from behavioral metrics (recall, clicks, and bookings).

Concisely, the same attributes that make the method thus far fast, open, and pragmatic justify a viable research agenda: scale and stratify; diversify media and contexts; stress-test analytic decisions; incorporate calibrated cross-ad anchors; connect attention shift to consequence; model temporal flow; expand segmentation for equity; open tooling; and experimentally manipulate key design levers. Seeing these through will convert an actionable descriptive atlas to an established, generalizable model for creating public-facing communications that reliably mobilize first glance into informed action.

## Figures and Tables

**Table 1:** Sample profile.

Characteristic		N	Percentage
Age group	40–45	9	30.0**%**
	46–50	5	16.7**%**
	51–55	7	23.3**%**
	56–60	9	30.0**%**
Household type	Married with children	19	63.3**%**
	Married without children	3	10.0**%**
	Single with children	3	10.0**%**
	Single without children	5	16.7**%**
Education	Primary school	4	13.3**%**
	Compulsory high school	13	43.3**%**
	University	7	23.3**%**
	Master’s degree and above	6	20.0**%**

**Table 2:** AOI descriptions and examples.

AOI Category	Description
Text	Headlines, body text, or captions
Symbol	Ribbons, hearts, symbolic elements (e.g., breast/statistics icons)
Image/Visual	Photographs, illustrations, or drawings
Logo	Corporate or organizational logos
Website	Stylized URLs or web references
Source/Authority	Institutions, endorsing bodies, or political figures

**Table 3:** Summary of aggregated AOI metrics.

Ad.	AOI	AOI Category	Mean FC (n)	Median TTFF (ms)	Median FD (ms)
Ad1	Ad1_WomansFace	Image	8.3	143.12	123.13
Ad1	Ad1_Icon(ribbon)	Symbol	2.06	634.84	462.24
Ad1	Ad1_HeadText	Text	27.59	297.05	206.86
Ad1	Ad1_Icon(heart)	Symbol	9.36	301.23	335.66
Ad2	Ad2_Icon(heart)	Symbol	10.61	821.84	318.78
Ad2	Ad2_HeadText	Text	2.82	458.08	224.83
Ad2	Ad2_Family	Image	18.51	433.11	207.17
Ad2	Ad2_Icon(breasts)	Symbol	18.83	111.16	299.67
Ad2	Ad2_Text	Text	3.23	299.89	224.68
Ad2	Ad2_FofiGenimata	Source/Authority	7.53	78.92	184.04
Ad2	Ad2_YpourgioYgeias	Source/Authority	2.41	508.57	221.19
Ad2	Ad2_Kivernisi	Source/Authority	22.73	280.79	205.72
Ad3	Ad3_HeadText	Text	10.6	123.45	277.23
Ad3	Ad3_Icon(ribbon)	Symbol	2.53	112.6	240.58
Ad3	Ad3_Icon1Statistics	Symbol	21.81	538.07	198.74
Ad3	Ad3_SmallText1	Text	12.63	372.69	250.69
Ad3	Ad3_Icon2Statistics	Symbol	2.16	299.57	187.76
Ad3	Ad3_SmallText2	Text	21.45	193.88	193.84
Ad3	Ad3_Icon3Statistics	Symbol	10.13	352.98	308.84
Ad3	Ad3_SmallText3	Text	2.23	961.78	217.58
Ad3	Ad3_BusinessWesbsite(Ygeia)	Source/Authority	20.93	98.67	220.25
Ad3	Ad3_BusinessLogo(Ygeia)	Logo	15.71	157.89	346.89
Ad4	Ad4_Picture(womanhand-drawn)	Image	6.05	171.52	486.3
Ad4	Ad4_HeadText	Text	2.16	440.88	463.63
Ad4	Ad4_Icon(breast)	Symbol	29.55	496.68	463.4
Ad4	Ad4_Text	Text	9.61	601.52	237.21
Ad4	Ad4_BusinessWebsite(AlphaBank)	Website	7.45	32.52	187.21
Ad4	Ad4_BusinessLogo(AlphaBank)	Logo	12.36	245.7	354.88
Ad5	Ad5_HeadText	Text	10.56	403.93	235.88
Ad5	Ad5_Picture(magnifier/breasts)	Image	2.86	324.59	203.79
Ad5	Ad5_Text	Text	15.43	489.78	173.55
Ad5	Ad5_BusinessWebsite(ProtoTheme)	Website	13.2	331.81	178.45
Ad5	Ad5_BusinessLogo(ProtoThema)	Logo	6.2	255.91	240.44
Ad6	Ad6_Headtext	Text	16.81	75.85	273.37
Ad6	Ad6_Icon(ribbon)	Symbol	23.3	231.66	211.28
Ad6	Ad6_picture(fist)	Image	13.95	90.45	212.45
Ad6	Ad6_IconswithText	Text	12.32	42.01	294.1
Ad6	Ad6_Website	Website	2.87	37.12	196.83
Ad6	Ad6_FofiGenimata	Source/Authority	16.49	76.11	202.77
Ad6	Ad6_YpourgioYgeias	Source/Authority	2.21	63.21	153.21
Ad6	Ad6_Kivernisi	Source/Authority	15.21	53.2	743.23

**Table 4:** First fixation percentage and median TTFF (ms) by AOI category and ad.

Ad	AOI Category	First-Hit %	Median TTFF (ms)
Ad1	Image	33.3	29
	Symbol	63.3	188
	Text	3.3	53
Ad2	Image	6.7	52
	Source/Authority	50.0	33
	Symbol	6.7	58
	Text	36.7	27
Ad3	Logo	13.3	18
	Source/Authority	6.7	51
	Symbol	50.0	30
	Text	30.0	41
Ad4	Image	30.0	2906
	Logo	10.0	137
	Source/Authority	0.0	48
	Symbol	20.0	194
	Text	16.7	55
	Website	23.3	18
Ad5	Image	36.7	31
	Logo	16.7	296
	Symbol	3.3	29
	Text	30.0	41
	Website	13.3	1187
Ad6	Image	0.0	203
	Logo	0.0	45
	Source/Authority	43.3	44
	Symbol	26.7	3565
	Text	16.7	332
	Website	13.3	284

**Note**. First-hit % is the share of participants whose first fixation landed on the category within each ad. Median TTFF reflects the median latency to first fixation among fixators of that category within the ad. Category presence varies by creative; large TTFF values (e.g., Ad4 Image, Ad5 Website, Ad6 Symbol) reflect delayed access to those categories in the competitive layout context.

**Table 5:** Top and bottom AOIs by dwell dominance (per ad). Values are dominance scores $S\;\in \;\left[-1,\;1\right]$.

Ad	Top-3 AOIs (S)	Bottom-3 AOIs (S)
Ad1	WomansFace (0.20) Icon(ribbon) (0.08) HeadText (−0.13)	Icon(heart) (−0.14) HeadText (−0.13) Icon(ribbon) (0.08)
Ad2	Text (0.38) Icon(heart) (0.23) FofiGenimata (0.13)	Icon(breasts) (−0.35) Family (−0.21) Kivernisi (−0.13)
Ad3	BusinessWesbsite(Ygeia) (0.22) BusinesLogo(Ygeia) (0.20) HeadText (0.12)	SmallText3 (−0.22) Icon(ribbon) (−0.15) Icon3Statistics (−0.10)
Ad4	Text (0.20) Picture(womanhand-drawn) (0.08) BusinessLogo(AlphaBank) (0.03)	HeadText (−0.16) BusinessWebsite(AlphaBank) (−0.13) Icon(breast) (−0.03)
Ad5	Picture(magnifier/breasts) (0.11) Text (0.08)	BusinessLogo(ProtoThema) (−0.08) HeadText (−0.07) BusinessWebsite(ProtoTheme) (−0.04)
Ad6	Kivernisi (0.22) Website (0.17) FofiGenimata (0.12)	Icon(ribbon) (−0.16) Headtext (−0.13) picture(fist) (−0.13)

**Note**. For ads with four or five AOIs, some “Top-3/Bottom-3” sets may share items when ranks are near the center; the narrative highlights the clearest winners and losers.

**Table 6:** Category-level dwell dominance scores by ad (−1...+1). Higher values indicate the category that “wins” more pairwise dwell contests within the ad.

Ad	Image	Symbol	Text	Logo	Website	Source/Authority
Ad1	−0.16	0.56	−0.40	—	—	—
Ad2	−0.65	−0.08	0.19	—	—	0.54
Ad3	—	0.47	0.52	−0.49	—	−0.49
Ad4	−0.29	−0.31	0.41	−0.28	−0.19	0.67
Ad5	−0.10	−0.20	0.72	0.02	−0.43	—
Ad6	−0.53	−0.18	0.47	0.00	−0.37	0.61

**Note**: Dashes “—” indicate categories not present for that stimulus.

**Table 7:** First-fixation percentage by AOI category and age group (per Ad).

Ad	Age	Image	Symbol	Text	Logo	Website	Source/Authority
Ad1	40–45	22.2	77.8	—	—	—	—
	46–50	60.0	40.0	—	—	—	—
	51–55	42.9	57.1	—	—	—	—
	56–60	22.2	66.7	11.1	—	—	—
Ad2	40–45	—	11.1	44.4	—	—	44.4
	46–50	20.0	20.0	20.0	—	—	40.0
	51–55	—	—	57.1	—	—	42.9
	56–60	11.1	—	22.2	—	—	66.7
Ad3	40–45	—	33.3	44.4	11.1	—	11.1
	46–50	—	40.0	40.0	20.0	—	—
	51–55	—	71.4	14.3	—	—	14.3
	56–60	—	55.6	22.2	22.2	—	—
Ad4	40–45	33.3	22.2	22.2	11.1	11.1	—
	46–50	20.0	40.0	—	—	40.0	—
	51–55	28.6	14.3	28.6	14.3	14.3	—
	56–60	33.3	11.1	11.1	11.1	33.3	—
Ad5	40–45	33.3	11.1	33.3	11.1	11.1	—
	46–50	40.0	—	20.0	40.0	—	—
	51–55	28.6	—	42.9	14.3	14.3	—
	56–60	44.4	—	22.2	11.1	22.2	—
Ad6	40–45	—	33.3	22.2	—	11.1	33.3
	46–50	—	—	40.0	—	20.0	40.0
	51–55	—	14.3	14.3	—	14.3	57.1
	56–60	—	44.4	—	—	11.1	44.4

**Note**. Values are the percentage of participants in each age stratum whose first fixation fell within each AOI category for the specified ad. Dashes indicate that no observations were available for that cell. Percentages are rounded to one decimal.

**Table 8:** First-fixation percentage by AOI category and household composition (per Ad).

Ad	Household	Image	Symbol	Text	Logo	Website	Source/Authority
Ad1	Married w/Kids	42.1	52.6	5.3	—	—	—
	Married w/o Kids	33.3	66.7	—	—	—	—
	Single w/Kids	33.3	66.7	—	—	—	—
	Single w/o Kids	—	100.0	—	—	—	—
Ad2	Married w/Kids	10.5	5.3	26.3	—	—	57.9
	Married w/o Kids	—	—	33.3	—	—	66.7
	Single w/Kids	—	33.3	66.7	—	—	—
	Single w/o Kids	—	—	50.0	—	—	50.0
	Single (no kids)	—	—	100.0	—	—	—
Ad3	Married w/Kids	—	52.6	26.3	10.5	—	10.5
	Married w/o Kids	—	66.7	33.3	—	—	—
	Single w/Kids	—	33.3	33.3	33.3	—	—
	Single w/o Kids	—	25.0	50.0	25.0	—	—
	Single (no kids)	—	100.0	—	—	—	—
Ad4	Married w/Kids	31.6	26.3	5.3	15.8	21.1	—
	Married w/o Kids	—	—	66.7	—	33.3	—
	Single w/Kids	33.3	—	33.3	—	33.3	—
	Single w/o Kids	25.0	25.0	25.0	—	25.0	—
	Single (no kids)	100.0	—	—	—	—	—
Ad5	Married w/Kids	31.6	5.3	31.6	15.8	15.8	—
	Married w/o Kids	—	—	33.3	66.7	—	—
	Single w/Kids	66.7	—	—	—	33.3	—
	Single w/o Kids	75.0	—	25.0	—	—	—
	Single (no kids)	—	—	100.0	—	—	—
Ad6	Married w/Kids	—	36.8	10.5	—	—	52.6
	Married w/o Kids	—	—	66.7	—	—	33.3
	Single w/Kids	—	—	—	—	66.7	33.3
	Single w/o Kids	—	—	25.0	—	50.0	25.0
	Single (no kids)	—	100.0	—	—	—	—

**Note**. Values are the percentage of participants in each household stratum whose first fixation fell within each AOI category for the specified ad. Dashes indicate no observations.

**Table 9:** First-fixation percentage by AOI category and education (per Ad).

Ad	Education	Image	Symbol	Text	Logo	Website	Source/Authority
Ad1	Compulsory/HS	38.5	53.8	7.7	—	—	—
	University	57.1	42.9	—	—	—	—
	Masters+	16.7	83.3	—	—	—	—
	Primary	—	100.0	—	—	—	—
Ad2	Compulsory/HS	7.7	—	23.1	—	—	69.2
	University	—	14.3	57.1	—	—	28.6
	Masters+	—	16.7	50.0	—	—	33.3
	Primary	25.0	—	25.0	—	—	50.0
Ad3	Compulsory/HS	—	30.8	53.8	7.7	—	7.7
	University	—	85.7	—	14.3	—	—
	Masters+	—	33.3	33.3	16.7	—	16.7
	Primary	—	75.0	—	25.0	—	—
Ad4	Compulsory/HS	23.1	30.8	7.7	7.7	30.8	—
	University	28.6	—	42.9	—	28.6	—
	Masters+	33.3	33.3	16.7	—	16.7	—
	Primary	50.0	—	—	50.0	—	—
Ad5	Compulsory/HS	30.8	—	30.8	23.1	15.4	—
	University	28.6	—	42.9	14.3	14.3	—
	Masters+	50.0	16.7	33.3	—	—	—
	Primary	50.0	—	—	25.0	25.0	—
Ad6	Compulsory/HS	—	30.8	7.7	—	15.4	46.2
	University	—	42.9	14.3	—	14.3	28.6
	Masters+	—	16.7	50.0	—	16.7	16.7
	Primary	—	—	—	—	—	100.0

**Note**. Dashes indicate no observations.

**Table 10:** Salient subgroup deviations in first-hit percentage (qualitative summary).

Ad	Age (vs. Overall)	Household (vs. Overall)	Education (vs. Overall)
Ad1	40–45 → Symbols/Images ↑	With children → Images ↑	University/Postgrad → Text ↑
Ad2	56–60 → Source/Authority ↑	With children → Family Image ↑	University/Postgrad → Text/Website ↑
Ad3	46–55 → Text/Statistics ↑	No children → Text/Website ↑	University/Postgrad → Statistics/Website ↑
Ad4	40–50 → Symbols/Image ↑	With children → Image ↑	University/Postgrad → Text ↑
Ad5	40–45 → Image ↑	With children → Image ↑	University/Postgrad → Text/Website ↑
Ad6	56–60 → Source/Authority ↑	No children → Website/Text ↑	University/Postgrad → Website/Text ↑

**Note**. Entries list the AOI categories showing the clearest positive subgroup–overall deltas in the first-hit heatmaps within each ad. Patterns are descriptive and intended to inform creative iteration rather than population-level generalization. “**↑**” = higher first-hit share than the ad’s overall. A slash “/” separates multiple AOIs with the same direction; the trailing arrow applies to all listed AOIs.

**Table 11:** Robustness checks for pairwise matrices.

Matrix/Scope	$\mathrm{Symmetry}\;P\;+\;P^{⊤}=\;1$	$\mathrm{Diagonal}\;P_{ii}\;=\;0.5$	Notes
AOI dwell-dominance (all ads, pooled)	Pass	Pass	Ties coded 0.5; low-N cells masked in figures
Category TTFF precedence (all ads, pooled)	Pass	Pass	“Earlier” is a win in precedence
Ad1 (AOI dwell dominance)	Pass	Pass	
Ad2 (AOI dwell dominance)	Pass	Pass	
Ad3 (AOI dwell dominance)	Pass	Pass	
Ad4 (AOI dwell dominance)	Pass	Pass	
Ad5 (AOI dwell dominance)	Pass	Pass	
Ad6 (AOI dwell dominance)	Pass	Pass	

**Note**. “Pass” indicates that the property is held to numerical tolerance in the computed matrices. Cells with support N < 5 were masked in visualizations but were not needed for these algebraic checks.

**Table 12:** Concordance between AOI dwell-dominance scores and median fixation duration (ms).

Ad	$n_{\mathrm{AOIs}}$	Pearson r	Spearman ρ
Ad1	4	0.81	0.80
Ad2	8	0.98	1.00
Ad3	10	0.87	0.96
Ad4	6	0.81	0.77
Ad5	5	0.68	0.50
Ad6	8	0.90	0.74

**Note**. Positive moderate-to-strong coefficients across ads indicate that AOIs that “win” pairwise dwell comparisons also tend to exhibit longer median dwell, supporting the substantive validity of the dominance index.

**Table 13:** Per-ad action guide from early-vs.-sticky quadrants.

Ad	Protect (Early + Sticky)	Promote (Late + Sticky → Move Earlier)	Unclutter (Early + Not Sticky)	Reconsider (Neither)
Ad1	Ribbon	Woman’s face	—	Headline; Heart icon
Ad2	Text body	Heart icon	Headline band	Family image; Breasts icon
Ad3	— (head text is modest+)	Website tag; Logo	—	Ribbon; SmallText3
Ad4	Hand-drawn image	Long text block	Website band	Headline
Ad5	Magnifier image	Central text	Headline	Logo; Website tag
Ad6	Government label; Website strip	— (endorser already early)	Ribbon	Fist image; Headline

**Table 14:** Atlas heuristics, from recurring pattern to practical fix.

Recurring Pattern	Evidence (Ads/Examples)	Actionable “Fix-It” Heuristic
Text/CTA late but sticky	Ad3 headline; Ad4 long text	Increase typographic contrast/scale; place in the vicinity of entry icon/image; dampen ambient competing salience
Icons outcompete text for entry	Ad1 ribbon; Ad5 magnifier	Use icons as portals (nest or arrow to CTA); balance icon weight to prevent CTA from being isolated
Logos/website sticky but found late	Ad3 website/logo; Ad4 copy	Shift towards entry path; introduce contrast edges (scale/weight/outlining); align on path of intuitive scan
Authority cues dominate in authority-forward layouts	Ad2, Ad6 labels/endorsers	Maintain CTA adjacency (space/micro-contrast); make CTA clear and legible
Decorative images early but not sticky	Ad4 website band; Ad5 headline visual pair	Slice or reuse decorative salience; introduce functional micro-copy to convert entry to dwell
Family imagery captures family households	Ad2 family image	Where there is the need for household targeting, otherwise down-weight to prevent draining the CTA.

**Note**. Patterns are derived from per-ad AOI rankings, early-vs.-sticky quadrants, and category transitions.

## Data Availability

The data presented in this study are available upon request from the corresponding author.
